# Current Diagnostic Techniques for Pneumonia: A Scoping Review

**DOI:** 10.3390/s24134291

**Published:** 2024-07-01

**Authors:** Kehkashan Kanwal, Muhammad Asif, Syed Ghufran Khalid, Haipeng Liu, Aisha Ghazal Qurashi, Saad Abdullah

**Affiliations:** 1 College of Speech, Language, and Hearing Sciences, Ziauddin University, Karachi 75000, Pakistan; 2 Faculty of Computing and Applied Sciences, Sir Syed University of Engineering and Technology, Karachi 75300, Pakistan; muasif@ssuet.edu.pk; 3 Department of Engineering, Faculty of Science and Technology, Nottingham Trent University, Nottingham B15 3TN, UK; 4 Research Centre for Intelligent Healthcare, Coventry University, Coventry CV1 5FB, UK; haipeng.liu@coventry.ac.uk; 5 Jubilee Healthcare Centre, Coventry CV1 3GB, UK; a.qurashi@nhs.net; 6 School of Innovation, Design and Engineering, Mälardalen University, 721 23 Västerås, Sweden

**Keywords:** diagnostic radiography, medical diagnosis, community-acquired pneumonia, COVID-19, non-invasive measurements

## Abstract

Community-acquired pneumonia is one of the most lethal infectious diseases, especially for infants and the elderly. Given the variety of causative agents, the accurate early detection of pneumonia is an active research area. To the best of our knowledge, scoping reviews on diagnostic techniques for pneumonia are lacking. In this scoping review, three major electronic databases were searched and the resulting research was screened. We categorized these diagnostic techniques into four classes (i.e., lab-based methods, imaging-based techniques, acoustic-based techniques, and physiological-measurement-based techniques) and summarized their recent applications. Major research has been skewed towards imaging-based techniques, especially after COVID-19. Currently, chest X-rays and blood tests are the most common tools in the clinical setting to establish a diagnosis; however, there is a need to look for safe, non-invasive, and more rapid techniques for diagnosis. Recently, some non-invasive techniques based on wearable sensors achieved reasonable diagnostic accuracy that could open a new chapter for future applications. Consequently, further research and technology development are still needed for pneumonia diagnosis using non-invasive physiological parameters to attain a better point of care for pneumonia patients.

## 1. Introduction

Pneumonia is the leading cause of mortality among infectious diseases. Despite thriving healthcare systems, pneumonia remains a serious health problem, causing 3.2 million of the 56.4 million deaths globally in 2015 [1]. Between 2000 and 2015, global hospital admissions for child pneumonia increased by 2.9 times, with a more rapid increase observed by the WHO in South East Asia than in African regions [2]. Pneumonia, though both preventable and manageable, is still a serious concern, especially for children under five years of age, and according to an estimate, 20% of pediatric deaths are caused by this vicious disease [3]. The early diagnosis and treatment of the disease should be considered a topmost priority, because pneumonia in children and elderly patients may lead to long-lasting effects on the lungs and the development of restrictive and obstructive lung function deficiencies [4].

**Figure 1:**
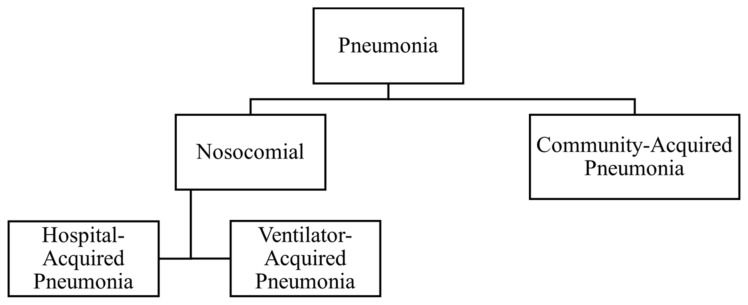
Classification of pneumonia according to the site of infection contraction.

Pneumonia is an infection of the lower respiratory tract. Mackenzie and Chang defined pneumonia as an acute infection of the lung parenchyma by various pathogens, excluding the condition of bronchiolitis [5]. Pneumonia can be classified according to the severity of the disease, as well as the site where the infection was caught, as shown in Figure 1. Community-acquired pneumonia is defined as pneumonia caught outside the hospital [6]. Nosocomial infection refers to infection contracted in hospital settings; hence, nosocomial pneumonia is further divided into hospital-acquired pneumonia (HAP) and ventilator-acquired pneumonia (VAP). Pneumonia that develops 48 h after a patient has been hospitalized is HAP, whereas VAP is defined as pneumonia that develops 48 h after intubation [7].

Various pathogens, including viruses, bacteria, and fungi, are known to cause pneumonia. The etiology of pneumonia is classified usually into typical bacteria, atypical bacteria, or respiratory viruses [6]. Atypical bacteria include Chlamydia pneumoniae, Mycoplasma pneumoniae, and Legionella species. Typical bacteria include *S. pneumoniae*, *H. influenzae*, *C. pneumoniae*, and *M. Pneumoniae*. Respiratory viruses include coronaviruses, respiratory syncytial viruses (RSVs), adenoviruses, influenza viruses, metapneumovirus, and parainfluenza viruses. Other causes include fungi, such as Histoplasma capsulatum.

Common clinical features according to age and severity of pneumonia are summarized in Table 1. The most common clinical symptoms of pneumonia include fever, dyspnea, fatigue, chills, cough, nausea, vomiting, diarrhea, and chest pain. Pneumonia is particularly risky for infants, children under the age of five, elderly over the age of 65, immunocompromised individuals, and patients with comorbidities. V. Averjanovaitė et al. found that COPD, comorbidities, and multilobar bilateral involvement are independent risk factors for severe early CAP complications [8]. Complications in CAP are common, and usually associated with delayed diagnosis of disease, misdiagnosis, or being given the wrong initial medication [9]. Accurately diagnosing pneumonia and differentiating it from upper respiratory tract infection and cardiovascular problems are important to rule out the unnecessary prescription of antibiotics. Antibiotic resistance has been observed in all pathogens associated with CAP. T. Welte et al. reported an increase in antibiotic-resistant strains; however, resistance was not related to mortality [10]. They found CAP to be associated with high hospitalization rates and length of hospital stay. They showed that the clinical and economic burden of CAP in Europe is high, that CAP has considerable long-term effects on quality of life, and that long-term prognosis is worse in patients with pneumococcal pneumonia.

Arthur. R. Reynolds published an article in 1903 that predicted pneumonia’s increased prevalence and pointed towards the need to restrict its spread [13]. He quoted Prof. William Osier, who termed pneumonia as the most widespread and fatal of all acute infectious diseases, stating that pneumonia is the “Captain of the Men of Death”. The statement still holds correct today, and the need to accurately and quickly diagnose pneumonia became even more evident with the recent outbreak of COVID-19 (SARS-CoV-2), which turned into a global pandemic. SARS-CoV-2 causes respiratory tract infection that progresses into systemic organ failure if not controlled [14]. We now have a few effective vaccines for COVID-19, and a reasonable portion of the world population has been vaccinated; however, humankind will keep encountering new strains of viruses that will make them vulnerable to respiratory infections. Hence, active research in diagnostic medicine into respiratory infections that cause pneumonia is highly needed.

In this paper, various current pneumonia diagnosis techniques in clinical settingsare reviewed. We undertook a scoping review to answer the following research objectives:

- RQ1: How many major categories are there for pneumonia diagnostic techniques?
- RQ2: What samples (body fluids or signals) have been used for each category and what techniques have been explored so far?
- RQ3: What is the possible course of action for enhancing the current state of the art for pneumonia diagnosis?

The rest of the paper is organized as follows: Section 2 discusses the methodology employed to select the research to answer the above-stated questions. Section 3 presents the results, and in Section 4, we briefly discuss them. Finally, in Section 5, we conclude the paper.

## 2. Materials and Methods

### 2.1. Study Design

The framework employed for the research design was that of a scoping review, as described by [15,16,17]. Two investigators independently performed the database search and screened the articles for relevance and whether they should be included. Any conflict was resolved by consensus.

### 2.2. Identification of Relevant Studies

Three electronic databases, namely IEEE Xplore, PubMed, and Science Direct, were searched using the keywords of “Pneumonia” AND (diagnosis OR detection OR screening) for the given scoping review, and a filter was applied to narrow the search to the years 2011 to August 2023. The initial search resulted in 773, 69,669, and 113,906 articles from the e-databases, respectively.

**Figure 2:**
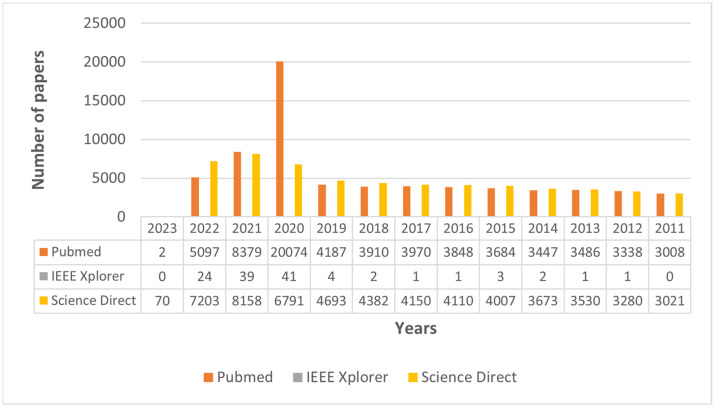
Number of journal publications in the major databases for the selected keywords for the defined time range.

Later, the search was limited to only journal articles, and 119, 66,430, and 57,068 papers were found, respectively. Only two very relevant conference papers were included after careful consideration by the authors. The year-wise distribution of the research article is shown in Figure 2. The first 1000 and 10,000 articles could not be accessed via Science Direct and PubMed only, as per database restrictions. Nine hundred and fifty-two (952) duplicates were removed using EndNote Web 20

### 2.3. Selection of Articles

#### 2.3.1. Data Screening

**Figure 3:**
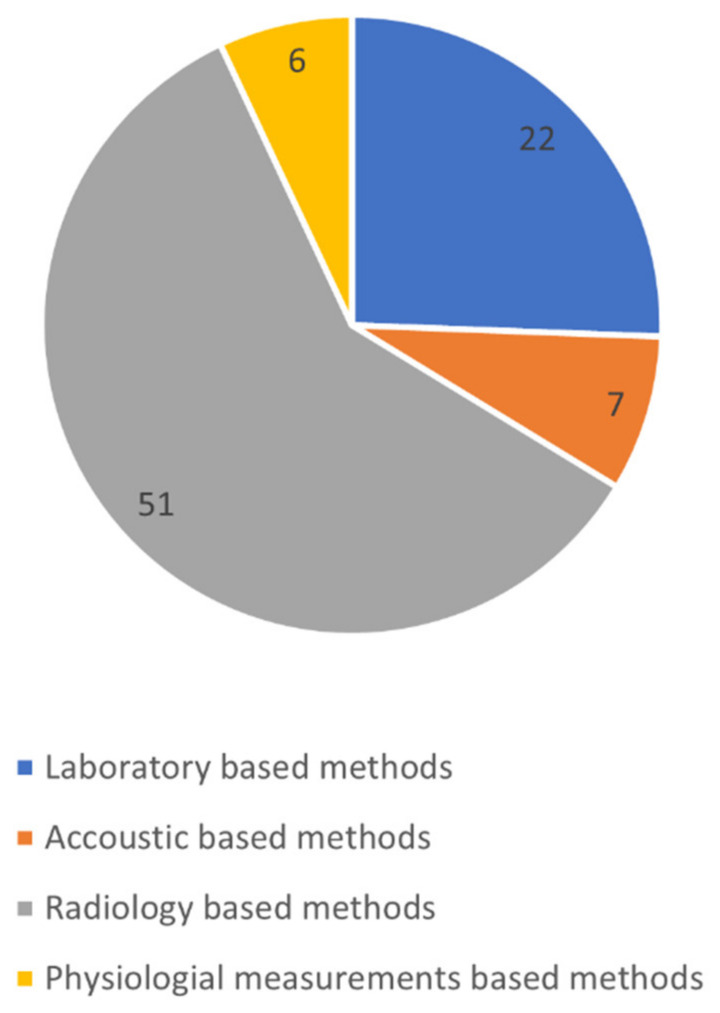
Number of papers for each technique included in the study.

**Figure 4:**
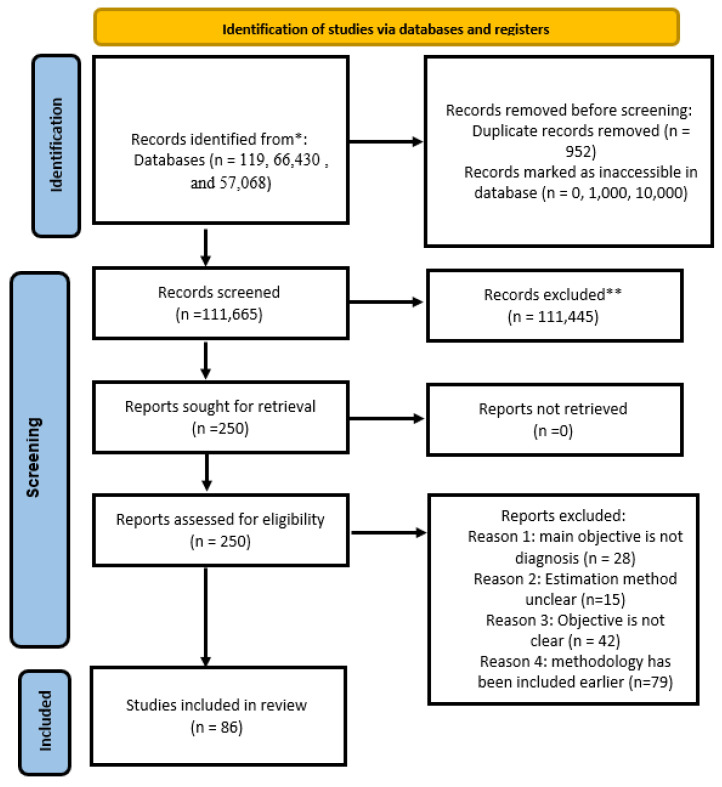
PRISMA 2020 flow diagram for selection of articles for the proposed scoping review. * IEEE Xplore, PubMed, and Science Direct, respectively. ** Duplicate records removed using EndNote Web 20.

The articles were then shortlisted by manual scrutiny using an inclusion criterion, and 86 publications were selected for this study. Figure 3 shows the distribution of papers for each technique incorporated in the study. Figure 4 shows the PRISMA flow diagram for the selection of articles.

#### 2.3.2. Inclusion and Exclusion Criteria

The following inclusion criteria were used to extract relevant studies:

1. Studies published between 2011 and 2023 in English.
2. Studies related to the diagnosis of pneumonia.
3. Peer-reviewed publications, preferably in a journal.

The exclusion criteria were as follows:

1. Methods not about the diagnosis of pneumonia.
2. Studies published before 2011.
3. Studies published in other languages.
4. Studies without any validation of proposed methods.
5. The estimation method is not properly defined.
6. Objectives are not mentioned.
7. Reviews, patents, editorial papers, surveys, technical reports, etc., are not included.

### 2.4. Data Charting

The publications were summarized for techniques, datasets used, evaluation methods, and results, and then categorized and compared according to modality or technique used to detect pneumonia using Excel spreadsheets and the Mendeley Cite citation manager. 

### 2.5. Summarizing and Reporting the Results

We categorized the research on the diagnosis of pneumonia into four categories using the scoping review framework. We focused on a broad range of studies and present a detailed overview, which are characteristic features of a scoping review [15]. We have provided a summary of the techniques used, the type of sample employed in each study, the datasets built or used where applicable, the evaluation method for generating the results, and a summary of the results with performance measures, where applicable, in the form of tables for each category. 

### 2.6. Patient or Public Involvement 

No patients or volunteers were involved.

## 3. Results

**Figure 5:**
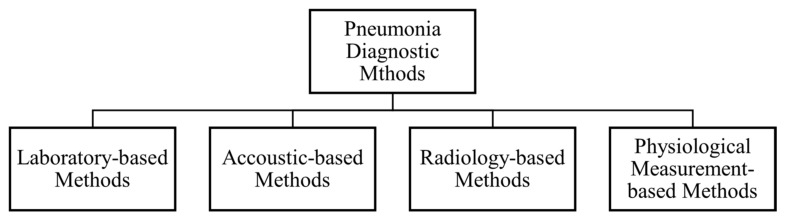
Pneumonia diagnostic methods.

As part of the review process, we categorized the pneumonia diagnostic methods into four classes, as shown in Figure 5. Each category is discussed in detail. Section 3.1 discusses laboratory-based diagnostic methods, Section 3.2 discusses acoustic- or chest-sound-based detection methods, Section 3.3 discusses imaging-based methods—within which CXR, CT and lung ultrasound methods are discussed individually—and finally, in Section 3.4, physiological-measurement-based methods are described. Additional papers are discussed in Section 3.5.

### 3.1. Laboratory-Based Diagnosis

**Figure 6:**
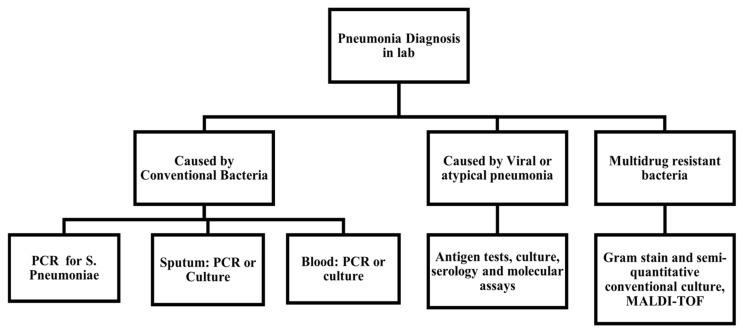
Possible lab-based tests for different causative agents, adapted from A. Torres et al. [18].

Table A1 presents the key publications carefully selected from the past ten years for laboratory-based methods, covering methods used, sample type used by each researcher, and evaluation techniques used to derive results and establish efficiencies. We have also summarized the data collected or generated by each work, and whether the results were compared with other techniques of CAP diagnosis. Finally, the key findings of each work are summarized under the results heading. Figure 6 presents a general overview of the techniques used in a laboratory setting for diagnosing various kinds of pneumonia [18]. The lab-based tests include a complete blood picture (CP, also known as complete blood count, or CBC), blood culture to rule out sepsis, and polymerase chain reaction (PCR)-based tests for viral pneumonia. C-reactive proteins (CRPs) are also greatly used since they indicate infection, but CRP values generally indicate infection alone, rather than pneumonia exactly. Clinicians sometimes prescribe antibiotics in the first week of infection under the wrong impression of raised CRP in viral infections [19]. Bronchoscopy and pleural fluid cultures are also used in severe undiagnosed cases.

### 3.2. Acoustic-Based Techniques

The method of diagnosing pneumonia using acoustic signals involves recording lung sounds with a digital stethoscope or similar device, then analyzing these sounds using advanced signal processing techniques and machine learning algorithms. Key features such as wheezes, crackles, and other abnormal respiratory sounds are extracted from the recordings. These features are then input into a trained classification model, which can distinguish between healthy and pneumonia-affected lungs. The process leverages the distinct acoustic patterns produced by pneumonia-related changes in the lungs, providing a non-invasive, quick, and cost-effective diagnostic tool that can be used in various settings, including remote or resource-limited areas. This method is undoubtedly the oldest and most hands-on technique practitioners use in clinical settings. Automatic auscultatory methods involve using digital devices such as microphones to pick up the acoustic signals from the chest. Such systems are usually used in the research phase, and no practical device is available. Such techniques have been particularly useful since teleclinics have been in practice, especially after COVID-19. A summary of papers using sound-based techniques for diagnosing pneumonia is presented in Table A2. The table summarizes the method and type of input sound used for diagnosis. It also briefly presents the evaluation techniques used to test the given method and the datasets used by each researcher. Finally, it can be extracted easily from the table if any given method has been compared with other related models or techniques, and accuracy, sensitivity, specificity, AUC, and other result parameters are given.

### 3.3. Imaging-Based Techniques

Imaging-based techniques such as CXR, CT scan, and LUS attempt to account for the anatomical changes occurring in the lungs due to pneumonia by taking physical images of the lungs. They can generally provide good insight, but are not feasible in limited-resource settings. The diagnosis of pneumonia is a combined result of correlating symptoms with lab results; however, the chest X-ray is considered the gold standard for diagnosing pneumonia [11,12,14,20].

**Figure 7:**
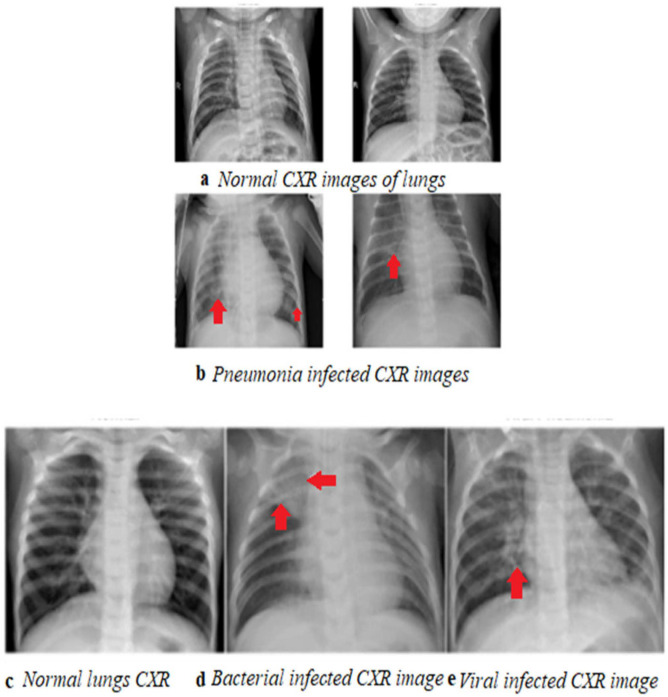
(**a**) Normal lung CXR images. (**b**) Pneumonia-infected lung CXR images taken from RSNA dataset. Bottom: CXR images of normal lungs, bacterial pneumonia, and viral pneumonia from left to right (**c**–**e**), taken from Kaggle, the open-access dataset [30,31].

Computed tomography (CT) scans are better in terms of accuracy; however, since they are generally not available in the primary care setting, CXR is preferred. CT is not a first-line imaging tool for uncomplicated community-acquired pneumonia. It is largely reserved for complications or difficulty differentiating CAP from other pathologies [21]. However, although not used much in practice, lung ultrasound (LUS) has recently been explored as an alternate option for diagnosing pneumonia instead of CXR or CT [22]. LUS could potentially be used instead of CXR, while the chest CT scan can be used for complicated cases [23]. LUS was found to be very sensitive (98.02%) and considerably specific (64.71%) for pneumonia originally confirmed with X-rays [24]. With the recent COVID-19 pandemic, a huge influx of knowledge on diagnosing pneumonia using both CXR and CT scans has been observed. Many attempts to diagnose CAP using LUS and MRI have been made [25,26]. CAP can be distinctively diagnosed based on any of the following three features or a combination of them: peribranchial nodules (bronchopneumonia), consolidation (alveolar/lobar pneumonia), or ground-glass opacity (GGO) [27]. The fourth unique and infrequent form is random nodules implying infection [28]. A glimpse of the work completed using CT scans to diagnose pneumonia in the past ten years is presented in Table A3. The role of CT scans in diagnosing COVID-19 has increased the development of many CT scan image databases. Because of their low resolution and fewer image details, it is very difficult to identify the difference between abnormal and normal lung CXRs, even for experienced radiologists and robust machine learning algorithms [29]. Typical CXR images with and without pneumonia taken from Kaggle and RSNA datasets are illustrated in Figure 7.

H. Kumarasinghe et al. employed a modified U-Net architecture for lung segmentation and an ensemble of CNN models for classification. Using the V7-labs COVID-19 X-ray dataset, the images were preprocessed, resized, and enhanced using CLAHE. Data augmentation techniques balanced the dataset. The modified U-Net included residual convolutional blocks and dropout layers, trained over 20 epochs with the ADAM optimizer and dice loss function. After the post-segmentation, lung areas were extracted and used to train the MobileNetV2, InceptionV3, ResNet50, and Xception models, which were combined into an ensemble classifier. The evaluation metrics included IoU score, dice coefficient, precision, and recall, demonstrating improved performance in lung segmentation and disease classification.

The methods for identifying pneumonia using CXRs, as well as the evaluation techniques and the datasets used by each researcher, are presented in Table A4. The table discusses if any given method has been compared with related techniques and, lastly, the results are summarized in terms of accuracy, sensitivity, specificity, AUC, and other parameters.

**Figure 8:**
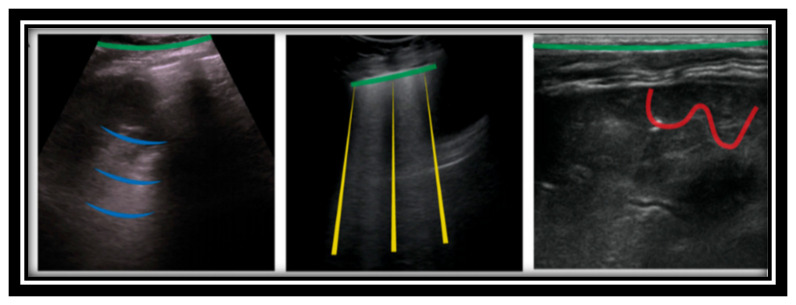
Reprinted: This shows the four types of lines found in LUS images. A lines are shown in blue; B lines are yellow; C line is shown in red; and the pleural line is green [33].

The key research articles presenting the lung ultrasound technique for diagnosing lower respiratory tract infection compared to CXR or CT scans are summarized in Table A5. The common pathological findings via LUS observed in the case of pneumonia are as follows: lines, hepatization, the shred sign, aerated bronchi, and hypoechoic parapneumonic effusion in the case of empyema [32]. Lung ultrasound images are shown in Figure 8. The pleura is seen as a hyperechoic horizontal line (green lines in Figure 8) [33]. The pleural line synchronizes with breathing, which is sometimes referred to as lung sliding. Additionally, below the pleural line, continuous hyperechoic horizontal lines appear as A lines (blue lines in Figure 8). Whenever aeration becomes compromised due to the accumulation of cells and fluids, the ultrasonic beam penetrates deeper into the lung, producing so-called B lines (comet-tail artefacts highlighted in yellow in Figure 8). The appearance of C lines indicates that lung consolidation has occurred as a result of an infectious pulmonary embolism, obstructive atelectasis, or a contusion of thoracic trauma (highlighted in red in Figure 8).

### 3.4. Physiological-Measurement-Based Techniques

A physiological-measurement-based system uses a number of proposed techniques to extract physiological parameters such as body temperature, respiratory rate, heart rate, and oxygen saturation via various sensors to diagnose pneumonia. Unfortunately, we only have such systems in the prototype phase. A summary of the work on this topic is presented in Table A6.

### 3.5. Results of Additional Papers

To perform a comprehensive review, a similar database search was performed limited to publications completed in 2024 only. T. Wanasinghe et al. presented a lung sound classification model on the ICBHI 2017 Challenge dataset, which contains respiratory sound recordings for 10 lung disease classes, utilizing a lightweight convolutional neural network (CNN) with multi-feature integration [34]. The proposed model integrates three audio feature representations—Mel-spectrogram, Mel-Frequency Cepstral Coefficients (MFCC), and Chromagram—into a stacked input in the CNN. The authors experimented with different CNN architectures like Xception, DenseNet, MobileNetV2, InceptionV3, ResNet50, and VGG16, and found that the stacked feature representation outperformed the others that used each feature individually. The authors evaluated the classification performance using accuracy, precision, recall, and F1 score metrics. They also used a weighted average approach to account for the imbalanced dataset. The proposed CNN model achieved the highest accuracy of 91.04% using the stacked feature representation, outperforming the individual-feature approaches. XAI analysis provided insights into the model’s decision-making process by highlighting the most contributive regions in the audio waveform. The authors conducted a comprehensive comparison of their work with existing studies in terms of feature selection, model architecture, datasets, number of classes, performance, and XAI techniques. They found that their approach achieved competitive results while also incorporating novel XAI analyses for audio data classification.

K. Kanwal et al. [35] used a machine learning-based approach to diagnose community-acquired pneumonia (CAP) in children using photoplethysmography (PPG) signals. The methods employed included the collection of PPG data from both healthy and pneumonia-infected children. A team of trained medical professionals under a consultant pediatrician diagnosed and labelled the participant PPG data with CAP or healthy tags. The PPG recordings were filtered, detrended, and normalized. Time- and frequency-domain features were extracted from the normalized PPG waves along with key points detected previously. The performance of the classifiers was evaluated using various metrics, such as accuracy, error, sensitivity, specificity, precision, F1 score, and area under the curve (AUC) for the receiver operator characteristic (ROC) curve. The dataset consisted of PPG signals from 57 participants (31 healthy and 26 pneumonia-infected) used for training and 10 participants (5 healthy and 5 pneumonia-infected) for testing. The highest accuracy was achieved by the linear discriminant classifier (84.09%), followed by the weighted KNN classifier (77.87%). The highest AUC-ROC value was associated with the linear discriminant classifier (0.82). The paper compares its results with existing studies in the field of respiratory rate estimation and pneumonia diagnosis using PPG signals.

## 4. Discussion

CAP stands globally as a lethal infectious disease, despite effective antibiotics and vaccines. The death rate for adult pneumonia as of 2018 was 93.2 deaths per 100,000 population for 65 year olds. The rate keeps increasing with age to as much as 377.6 per 100,000 population [36]. Pneumonia is a leading cause of hospitalization for both extreme age groups (elderly and infants) [37,38] and remains a challenging health issue owing to the evolving microbial world that keeps generating novel causative agents for pneumonia. Humankind has experienced avian flu, Middle East respiratory syndrome (MERS), severe acute respiratory system (SARS) coronavirus, and SARS-CoV-2, resulting in COVID-19-induced pneumonia. The threat of pandemics and endemics shall remain a serious problem. We are currently fighting one of the most lethal pandemics, resulting in 3.73 million deaths worldwide to date [39]. It is, therefore, important to devise accurate, time-efficient, and deterministic diagnostic techniques to detect pneumonia and differentiate it from other similar pathologies like congestive heart failure.

Previously, systematic reviews of a very specific nature have been completed for the diagnosis of ventilator-acquired pneumonia (VAP) and hospital-acquired pneumonia (HAP) [40,41]. The review papers for CAP are mostly directed towards the management of CAP [42]. For the diagnosis of CAP, review papers usually target a specific area or method of diagnosis, like biomarkers only, as seen in [43,44], or a specific imaging modality [45]. Hence, we have tried to bridge the gap by presenting a scoping review of the many techniques currently used to diagnose CAP. We have attempted to summarize the pros and cons of each method in the present work to make them easier to comprehend. Very recently, Stokes et al. completed a systematic review of AI-based models for diagnosing pneumonia using signs and symptoms [46]. E. Gentilotti et al. performed a systematic review and meta-analysis of the diagnostic accuracy of point-of-care tests, including lung ultrasounds, X-rays, rapid antigen tests, etc. [47]. A. Heidari et al. performed a systematic literature review for diagnosing COVID-19-induced pneumonia using deep learning methods [48]. In our opinion, these reviews represent only a small portion of diagnostic methods, and AI-based methods are not practiced in clinical settings yet. It was not a feasible option to perform a meta-analysis of different diagnostic methods with different key performance measures; hence, a scoping review has been completed so that we can underline future directions for research and advancement in the field.

This scoping review highlights the need for a comprehensive review that categorizes diagnostic methods into four classes: laboratory-based, acoustic- or chest-sound-based, imaging-based, and physiological-measurement-based. Many surveys focus on specific aspects of pneumonia diagnosis, such as imaging-based methods or laboratory tests, without providing a broad overview of the various techniques used. Some reviews are restricted to specific databases or periods, which can lead to incomplete or outdated information. Different reviews use varying inclusion and exclusion criteria, making it difficult to compare results across studies. Previous reviews have often failed to provide a detailed analysis of the techniques used, datasets employed, and evaluation methods, making it challenging to understand the strengths and limitations of each method. Existing reviews often group methods into broad categories without providing a detailed breakdown of the techniques used within each category.

To address these limitations, this scoping review paper presents a comprehensive scoping review that includes laboratory-based, acoustic- or chest-sound-based, imaging-based, and physiological-measurement-based methods, providing a detailed overview of the various techniques used in pneumonia diagnosis. Standardized inclusion and exclusion criteria were employed to ensure consistency and comparability across studies. The review includes detailed analyses of the techniques used, datasets employed, and evaluation methods, enabling a better understanding of the strengths and limitations of each method. We categorized methods into four classes and provide a detailed breakdown of the techniques used within each category, allowing for a more nuanced understanding of the methods. The review covers articles from IEEE Xplore, PubMed, and Science Direct, and includes studies published between 2011 and 2023, ensuring a comprehensive and up-to-date overview of the field. By addressing these limitations, this paper provides a novel contribution to the field of pneumonia diagnosis by presenting a comprehensive and detailed review of the various techniques used, which can help researchers and clinicians better understand the strengths and limitations of each method and inform the development of more effective diagnostic tools.

This paper presents a summary of the various techniques used to diagnose community-acquired pneumonia; to answer RQ1, we extracted research papers from three databases and divided them into four categories based on the techniques used for detection. We then described each method briefly and analyzed them in terms of techniques employed and results achieved to answer RQ2. In the section above, to answer RQ3, we have summarized the pros and cons of using each method. With the huge surge of information after the COVID-19 pandemic and concerns about infection spread, it is impossible to declare the single best method for pneumonia detection. Testing should be undertaken depending on the availability of resources and the type of care setup. Diagnostic tests should be encouraged for patients at greater risk, such as infants, the elderly, and immunocompromised individuals, since proactive testing is a key step for the rapid diagnosis and treatment of pneumonia. Given the various available methods, we understand it is impossible to make a conclusive statement about the best diagnostic method; hence, a meta-analysis was not completed. However, a class-wise division of diagnostic methods has been established and we have identified potential directions of future research for the better diagnosis of pneumonia in each class of technique.

**Figure 9:**
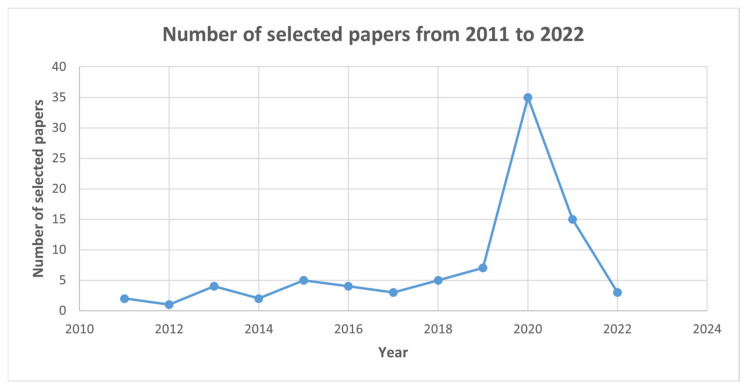
Number of selected studies in the given period. There was a surge in research containing the keyword pneumonia in 2020, and the trend continued after this point. This could be explained as being related to the COVID-19 pandemic.

Figure 9 represents a graph of the number of selected papers that were published during the selected time. It is evident that research containing the keyword of pneumonia had a surge in 2020. This could be a consequence of the outbreak of COVID-19-induced pneumonia. 

**Figure 10:**
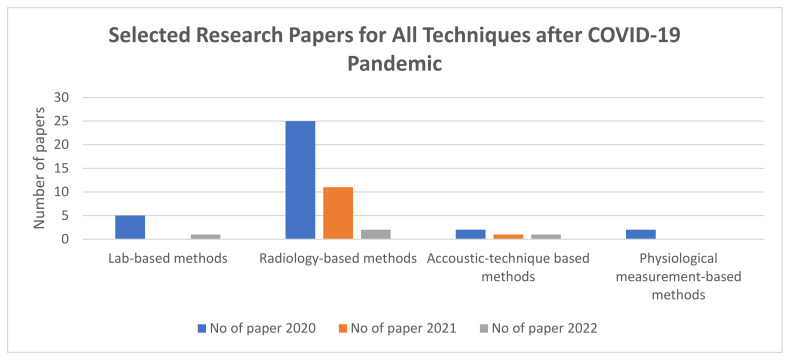
Technique-wise distribution of the number of selected papers for the time when research related to pneumonia had a surge. Radiology-based techniques were the most researched.

Figure 10 represents a technique-wise distribution of the selected papers for the year 2020 and the following years. During the years 2020, 2021, and 2022, there were 25, 11, and 2 selected papers that focused on radiology-based methods, respectively. This implies that most of the research during this period was geared towards radiology-based methods.

**Figure 11:**
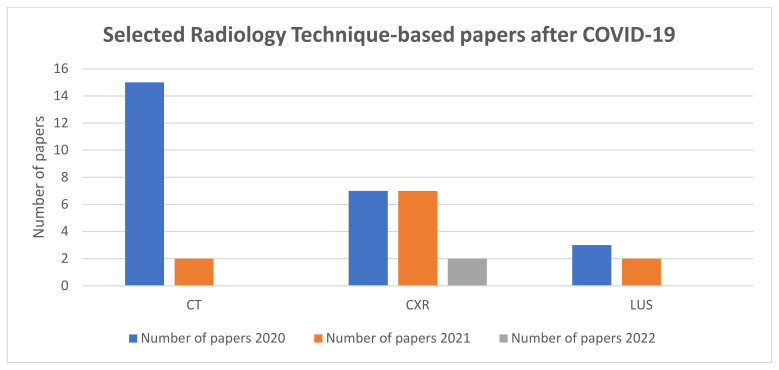
Categorical distribution of selected papers on radiology for the three years after the outbreak of COVID-19.

From Figure 11, it can be concluded that most of the researchers targeted CT scans and CXR for pneumonia diagnosis instead of other methods during this period.

**Figure 12:**
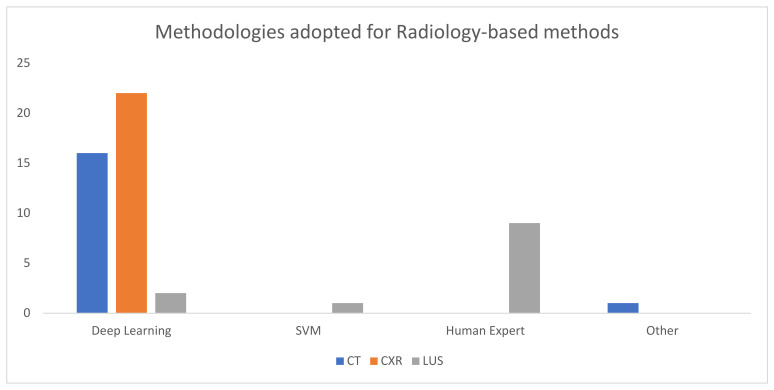
The methodologies employed for diagnosing pneumonia using radiographic images. Advancements in deep learning and machine learning have been a fundamental reason for targeting images.

Finally, in Figure 12, we have attempted to visualize the different methods adopted to diagnose pneumonia using radiology-based methods, and it can be observed that almost 33 papers on CT scans and CXR were based on a deep learning method. This implies that advancements in deep learning, machine learning, and related fields are a primary reason for the influx of knowledge related to this field.

The review presented in this paper could be improved by including more research databases and conference papers of good quality. The search was skewed by and includes more research related to COVID-19-induced pneumonia because of the plentiful efforts in the last three years by researchers around the globe to formulate reliable diagnostic techniques during the global pandemic. Many researchers have attempted to perform machine learning on radiographic images. Most of the papers considered in the present review cover imaging-based techniques. Lab-based techniques can be further divided into various types based on the type of sample or method of diagnosis employed. This could give a further understanding of the lab-based techniques currently in practice. Lastly, we have not attempted to perform any meta-analysis or statistical valuation to compare accuracy, sensitivity, or specificity because different performance measures were discussed in the included studies and the entire process was not expected to make any sense.

Lab-based tests are very sensitive and adaptable, and can detect the entire range of respiratory pathogens; however, they are generally time-consuming. Pathogen cultures often take many days and several target detection systems (or multiplex assays); in general, nucleic acid amplification by polymerase chain reaction (PCR) needs several hours to yield results. Certain biological compounds as biomarkers for disease can be employed to fabricate biosensors for the rapid diagnosis of pneumonia; however, we have limited research to support the claim. Lab-based techniques are currently the only way to find resistance patterns in pathogens causing pneumonia. Many diagnostic arrays and systems have been developed for rapid diagnosis, but the validation of new diagnostic technology platforms is crucial to evaluate their effectiveness and guide antibiotic treatment in this population.

Acoustic-based techniques are generally accurate and sensitive, but usually nonspecific. The practice of deep learning is expected to improve the specificity of using chest sounds for pneumonia diagnosis. Such systems can be better utilized for initial screening and monitoring, and should be used with other testing techniques for diagnosis. Most of the proposed systems are in the prototype phase and have not gone through clinical trials; thus, it is too early to expect any of the systems to be commercially available.

Deep learning-based image segmentation algorithms applied to CXR and CT scans are very accurate and can be used where available. However, they are computationally intensive. Preprocessing techniques need to be improved to decrease computational requirements at the detection phase. Point-of-care lung ultrasonography should be explored with the powerful computation ability of machine learning to achieve greater specificity. Using deep learning systems, researchers working with CXR images have achieved accuracy, sensitivity, and specificities up to 98%, 99%, and 96%. The downsides of deep learning are the huge computational requirements that make it unfeasible for primary health care centers and the unavailability of such systems as commercial products. CXR interpretation by technicians or physicians is prone to error, making it an unsuitable choice as the gold standard. The accuracy of CT scans is comparable with deep learning-based CXR algorithms with more specificity. With the development of public-access databases for CXR and CT images in the past two years, a huge improvement in the diagnosis of pneumonia type has been observed, and it is expected that new, improved algorithms can be made that will help discriminate between pneumonia types with greater accuracy. CT scan facilities are scarcer than those for CXRs. For example, given the population of 216.6 million in Pakistan, only 80 CT scanners are present, meaning that the demand of the general population cannot be met [49]. The better specificity offered by lung ultrasonography is promising, and we expect more utility of point-of-care ultrasonography for pneumonia detection in the coming years, with research being undertaken in the relevant field.

Advancements in medical technology have led to the development of several inexpensive, accurate, and easy-to-operate sensors and techniques that are used to extract valuable signals from the human body and measure physiological parameters. With the development of accurate non-invasive sensors and technologies, it is possible to formulate devices that can extract relevant physiological parameters useful in diagnosing pneumonia. Such techniques would not only decrease the cost of lab-based tests, but would be faster and more user-friendly. COVID-19 drew the attention of many researchers towards radiography. It is worth mentioning that the usage of simple sensors such as optical sensors can extract valuable information about both respiratory and cardiovascular systems, both of which carry information about pneumonia status. Current state-of-the-art hospitals, diagnostic facilities, and care centers should have the option of remote screening and monitoring for pneumonia, which can be made possible by using these techniques. G li et al. [50] proposed a personalized remote monitoring method based on body temperature and heart rate. They established a body area network for edge computing that builds a blockchain of COVID-19-infected individuals. Their objective was to ensure patient security. This confirms that the scientific world is shifting with technology, as imposed on us by the last pandemic. Our brief review of current techniques based on vital signs suggests that they are more for monitoring than diagnosis. The information about the respiratory and cardiovascular systems carried by biological signals encoded in PPG can be used for diagnostic purposes, and is an interesting research area. Applying machine learning on such signals could be very useful.

For real-world deployment, the proposed model shows promise due to its high accuracy in segmenting lung areas and classifying chest X-rays for COVID-19 and pneumonia. However, several challenges need to be addressed for it to be adopted in clinical practice. These include ensuring the robustness of the model across diverse patient populations and clinical settings, integrating the model into existing healthcare IT infrastructures, and complying with regulatory standards for medical devices [51]. Additionally, comprehensive validation through clinical trials would be necessary to demonstrate its effectiveness and safety in real-world scenarios [52,53]. Overcoming these hurdles could enable the deployment of this model as a valuable tool for aiding diagnosis and improving patient outcomes in clinical environments.

## 5. Conclusions

A comprehensive search and scoping review of the techniques available for diagnosing CAP are beneficial for clinical health workers and clinical engineers. A tremendous amount of research has been undertaken to diagnose and detect pneumonia in the past few years. This study has summarized all possible techniques for diagnosis and highlighted areas of further research and improvement. The automatic detection of pneumonia features using CXR, CT, and LUS images has been attempted using the strong ability of deep learning-based techniques to extract complicated features from data. Various review papers have been published targeting CXR- or LUS-based techniques for pneumonia detection, but a comprehensive guide on possible techniques has been lacking. Based on the present study, researchers can understand current techniques in practice, as well as their clinical significance, and attempt innovative techniques to overcome existing limitations. Acoustic-based and physiological-parameter-based techniques can be used to devise accurate, inexpensive, and POC devices for the diagnosis of pneumonia in the clinical setting, and this area should be explored further.

## Figures and Tables

**Table 1:** Clinical features of CAP summarized from [11,12].

Degree of Illness	Age Group		
	Infants	Older Children	Adults
Mild/Moderate	^1^ Temp: <38.5 °C ^2^ RR: <50 bpm Mild recession Normal feeding	^1^ Temp: <38.5 °C ^2^ RR: <50 bpm Mild dyspnea No vomiting	^1^ Temp > 39 °C ^2^ RR > 30 bpm Dyspnea Mild cough
Severe	^1^ Temp: >38.5 °C ^2^ RR: >70 bpm Mild-to-severe recession Respiratory distress Tachycardia Intermittent apnea Decreased feeding Capillary refill time > 2 s	^1^ Temp: >38.5 °C ^2^ RR: >50 bpm Mild-to-severe recession Respiratory distress Tachycardia Intermittent apnea Decreased feeding	^1^ Temp > 39 °C Respiratory distress Cough Low systolic blood pressure
Very severe	Cough or difficulty in breathing Oxygen saturation <90% or central cyanosis Severe respiratory distress (e.g., grunting, very severe chest indrawing) Signs of pneumonia with a general danger sign (inability to breastfeed or drink, lethargy or reduced level of consciousness, convulsions)		Temp > 40 °C RR > 30 bpm SpO_2_ < 92 Arterial pH < 7.5 Multiple organ dysfunctions Altered mental state Pleuritic chest pain Adventitious breath sounds

^1^: Temperature. ^2^: Respiratory rate.

## Data Availability

The data that support the findings of this study are available on request from the corresponding author.

## Appendix A

**Table A1:** Brief summary of key publications focusing on laboratory-based techniques for diagnosing pneumonia published in three major databases, namely IEEE Xplore, PubMed, and Science Direct. This table presents the method and sample type used by each researcher.

Author	Techniques	Sample Type	Evaluation Methods	Data	Comparison with Other Studies or Other Models	Results
Zhou Li et al. (Jan, 19) [54]	Biomarkers for pneumonia	ECG (electrocardiogram), serum levels of CK, CK-MB, and troponin (creatine kinase, creatine kinase-MB, and heart muscle enzyme)	Mean, standard deviation, one-way ANOVA, and pairwise comparison performed by *t*-test and (Chi-squared tests) χ2 test	Prospective study	No	CK, CK-MB, and troponin serum levels increased with the severity of the disease
Naomi J. Gadsby et al. (Apr, 16) [55]	Culture, RT-PCR (real-time polymerase chain reaction)	LRT specimens	Fisher exact test or χ2 test, Shapiro–Wilk W test, Mann–Whitney U test, and *t*-test	Patients presented to 2 tertiary care hospitals in Edinburgh (UK) over 18 months	Yes	A comprehensive molecular testing approach approximately doubles pathogen detection in patients with CAP from 39.3% to 86.7%
R. Borgohain et al. (Mar, 17) [56]	Novel ZnO Biosensor	Prepared solutions for the mentioned bacterium	Graphs of voltage and concentration, etc.	Not applicable	No	Maximum response = 96% and 94.375%, min detection limit = 1.12% and 1.01% @ room temperature
Madieke J. Koster et al. (Jul, 13) [57]	CRP level (C-reactive protein)	Blood samples	(Un)adjusted association between CRP level and pneumonia and the diagnostic value of CRP investigated	Retrospectively collected data from the ED (Antonius Hospital Nieuwegein)	Yes	Mean CRP children with pneumonia = 141 mg/L; without pneumonia = 34 mg/L
R. Sorde et al. (Jan 11) [58]	Urinary antigen test	Urine samples	Sensitivity, specificity, positive and negative predictive values, as well as positive and negative likelihood ratios	Adults hospitalized with CAP from February 2007 to January 2008	No	Positive predictive value 88.8% to 96.5%
S. L. Yang et al. (Oct, 20) [59]	RT-PCR	BAL (bronchoalveolar lavage) and sputum samples	Two-tailed Student’s *t*-test	97 BAL samples and 94 sputum samples from 191 patients were used in the study	No	Diagnosis of PCP (pneumocystis pneumonia) should be based on a combination of clinical symptoms, underlying diseases, and PCR (polymerase chain reaction) results due to FP (false positive) in PCR
Jan C Holter et al. (Feb, 15) [60]	Bacterial cultures, urinary antigen assays, serology compared with swabs	NP and OP swabs (nasopharyngeal and oropharyngeal swab)	McNemar’s test, kappa statistics	3-year prospective study, Drammen Hospital, Vestre Viken Health Trust	No	The ratio ranged from 14.6 to 19.9
A. Ito et al. (Feb, 21) [61]	Urinary antigen	Urine samples	Cross-tabulation table	Study in 6 hospitals in Japan in 32 months, approx.	Yes	The overall match rate between LAC-116 (urinary antigen test kits) and Binax was 96.8% and between LAC-116 and Q-line was 96.4%
Yuan Lu et al. (Dec, 11) [62]	PCR	URT (upper respiratory tract) specimen	AUC (area under ROC curve)	NA	No	Highest specificity of 0.93 between quantitative PCR analysis and the major surface glycoprotein gene target
M. A. Elemraid et al. (Jun 2013) [63]	Culture and PCR testing	Blood samples and other fluid samples	*p*-value analysis	From October 2009 to March 2011 in three hospitals in Northeast England	No	Pneumococcal infections identification rate = 26%. Detection improved with PCR compared to with culture
F. Esteves et al. (Apr, 15) [64]	Biomarkers for pneumonia	Serum	Chi-square test, Fisher’s exact test, Mann–Whitney U test, Spearman rank-order correlation test, ROC (receiver operating characteristic curve)	Retrospective observational study	Yes	Reliability of markers for PCP diagnosis: (1-3-Beta-D-Glucan) BG > (Krebs von den Lungen-6) KL-6 > (Lactate Dehydrogenase) LDH > (S-adenosylmethionine) SAM. Best combination test = BG/KL-6
Y. Jiang et al. (Dec, 20) [65]	Digital PCR	Patient and environmental samples	Comparison of positivity rate	Self-collected	Yes	SARS-CoV-2 was more frequently detected in respiratory tract-derived samples (35.0%) than in non-respiratory tract-derived samples
D. Xiao et al. (Aug, 12) [66]	MALDI-TOF MS (matrix-assisted laser desorption/ionization (MALDI)–time-of-flight (TOF) analyzer)	Throat swabs	Chi-square test	The prospective study included 70 CAP patients. Compared with Biotyper and SARAMIS databases	Yes	A total of 212 suspicious colonies representing 12 genera and 30 species were identified
Y. Wang et al. (Jun, 18) [67]	Multiple cross displacement amplification (MCDA)	Sputum	Accuracy, sensitivity	K. pneumoniae-negative sputa (five patients) collected from the Clinical laboratory of Peking University Shougang Hospital	Yes	A temperature of 65 °C was found to be optimal for amplification, and seven K. pneumoniae strains were successfully cultured from positive sputum samples
B. Medjo et al. (Dec, 14) [68]	RT-PCR and Serology	Throat swabs	Student’s *t*-test, Mann–Whitney, Chi-square test, and Fisher’s exact test	Prospective study Emergency department of University Children’s Hospital in Belgrade from April 2012 to March 2014	Yes	The detection of IgM antibodies in conjunction with 8RT-PCR allows for the accurate and reliable diagnosis of Mycoplasma pneumoniae infections in children at a critical stage
A. Edin et al. (Jul, 20) [69]	PCR panel	NP swabs	Two-by-two contingency tables of categorical variables were analyzed by Fisher’s exact and Wilson–Brown method	Conducted at Umeå University Hospital (Umeå, Sweden) as a prospective method-comparison study	Yes	In 30% of admitted patients, survey results were found to have an impact on treatment decisions
A. Banerjee et al. (Sep, 20) [70]	Machine learning	Blood samples	AUC, sensitivity, specificity, and accuracy	mindstream.ai public dataset	No	Changes in numerous parameters assessed in the complete blood count for COVID-19-positive individuals with a characteristic immune response profile pattern
Chih-Min Tsai et al. (Jul, 20) [71]	CRP as a biomarker	Saliva samples	Chi-squared test, *t*-test statistical correlations using Spearman’s rank correlation test, ROC	Prospective research was carried out on patients aged 2–17 years; 60 healthy children and 106 pediatric patients suffering from pneumonia	Yes	Salivary CRP level pediatric patients with pneumonia = 48.77 ± 5.52 ng/mL healthy = 14.78 ± 3.92 ng/mL (*p* < 0.001)
A Omran et al. (Apr, 18) [72]	CRP level, blood counts	Saliva and blood samples	Mann–Whitney U-test, Chi-square, sensitivity, specificity, ROC curve	Prospective case–control research included 70 full-term newborns, 35 with late-onset neonatal pneumonia, and 35 healthy controls	Yes	Salivary CRP means (neonates with late onset) = 6.2 ± 4.6 ng/L versus control neonates = 2.8 ± 1.9 ng/L
F. Patrucco (Mar,19) [73]	Pneumoni check^TM^	Aerosol	Sensitivity, specificity	A prospective single-center observational pilot study	Yes	Excellent specificity and correlation with 10BAL for non-herpes virologic diagnosis in pneumonia patients
M.C. Minnaard (Jul, 15) [74]	CRP level	Blood sample	AUC, multivariable odd ratios	GRACE network	Yes	Five POC CRP test tools and the lab analyzer detected pneumonia with comparable accuracy in a single test
Y Saffary et al. (July, 22)	Tetracosane functionalized TiO_2_ sensor	Expired breath (gas)	Mean, standard deviation	Not tested in vivo	No	The functionalized sensor showed a change in current when exposed to heptane. The sensor response was found to be varied with varying concentrations

**Table A2:** Summary of key research articles from the last ten years using acoustic techniques for the diagnosis of CAP, retrieved from IEEE Xplore, PubMed, and Science Direct. This table summarizes the methods and types of input sound used for diagnosis.

Author	Techniques	Input Parameters	Evaluation Methods	Data	Compared with Related Work	Results				
						Acc. (Accuracy)	Sen. (Sensitivity)	Spec. (Specificity)	AUC (Area Under ROC Curve)	Other
K. Kosasih et al. (Apr, 15) [75]	Wavelet features of cough sounds trained a logistic regression classifier	Cough sound	Sensitivity, specificity	815 cough sounds from 91 patients with respiratory illnesses	No	(Not reported) NR	0.94	0.88	NR	NR
H. Chen et al. (Mar, 19) [76]	Optimized S-transform (OST) and deep residual networks (ResNets),	Respiratory sound	Classification accuracy, confusion matrix, standard error, standard precision	Biomedical Health Informatics (ICBHI) scientific challenge database	Yes	0.9879	0.9627	1	NR	NR
P. Porter (Mar, 21) [77]	Smartphone-based algorithm	Cough sound	Percentage agreement (PA), AUC	A prospective cohort study in a hospital in Western Australia	No	NR	NR	NR	0.94–0.95	PPA = 86.2%, NPA = 86.5%
Eric D. McCollum et al. (Sep, 20) [78]	Random-effects regression model	Chest sounds	T-test, Pearson, χ2(Chi-squared test), or Fisher exact test. Multiple logistic regression	Prospectively enrolled hospital cases and community controls over two years in seven countries	Yes	Wheezing among children autonomously associated with lower odds of radiographic pneumonia				
A. Rao et al. (Aug, 18) [79]	K-nearest neighbor	Chest sounds	Accuracy, frequency spectrum	Clinical data collected from UCSF Medical Center	No	0.923	NR	NR	NR	NR
A. Imran et al. (2020) [80]	Distinctness of pathomorphological alterations in the respiratory system	Cough sound	Accuracy, specificity, sensitivity/recall, F1 score	ESC-50 dataset [62], self-collected via app	No	0.956	0.8914	0.9667	NR	F1 Score: 0.8952 precision 0.8991
RK Tripathy et al. (May 2022) [81]	Time and frequency features from sound, employed various classifiers for different classes	Chest sounds	Accuracy	M. Fraiwan et al. dataset [82]	Yes	Accuracy SVM: 80.35; Random Forest: 83.27; extreme gradient boosting: 99.34; and light gradient boosting machine (LGBM): 77.13%				

**Table A3:** Summary of key research articles from last ten years using CT scans (an imaging-based method for the diagnosis of CAP), retrieved from IEEE Xplore, PubMed, and Science Direct. This table summarizes the algorithms or methods used to diagnose pneumonia using CT scans. It also briefly presents the evaluation techniques used to test the given methods and the datasets used by each researcher. Finally, it can be extracted easily from the table if any given method has been compared with other related models or methods, and accuracy, sensitivity, specificity, AUC, and other result parameters have been given.

Author	Techniques	Evaluation Methods	Data	Compared with Related Work	Results					
					Acc. (Accuracy)	Sen. (Sensitivity)	Spec. (Specificity)	F1 Score	AUC (Area under ROC Curve)	Other
Yaoming Lai et al. (Oct, 2020) [83]	Multiscale deep convolutional neural network (DCNN)	ROC curve (receiver operating characteristic curve), accuracy, specificity, sensitivity, AUC	Two hospitals’ retrospective study data	Yes	0.861	0.757, 0.757	0.952, 0.815	(Not Reported) NR	5%	NR
G. Wang et al. (Aug, 2020) [84]	2D convolutional neural network for segmentation + COPLE net (COVID-19 pneumonia lesion segmentation network)	Paired T-test	Ten hospitals’ retrospective study data	Yes	NR	NR	NR	NR	NR	Dice % COPLE-NET 80.29+−11.14
Z. Wang et al. (Oct, 2020) [85]	Novel joint learning based on COVID-Net	*p* values for paired *t*-test	SARS-CoV-2 and COVID-CT data	Yes	NR	NR	NR	NR	12.16%, 14.23%	NR
Q. Wang et al. (May, 2020) [86]	Deep regression framework and RCNN (region-based convolutional neural network) based on ResNet-50	Accuracy, sensitivity, specificity, and F1 score	Radiology department dataset	Yes	Improves by 2.3%	Improves by 3.1%	NR	NR	BR	NR
H. Kang et al. (May, 2020) [87]	Backward neural networks	Accuracy, sensitivity, and specificity	Three hospitals’ and collaborator’s study data	Yes	0.95	0.966	0.932	NR	NR	NR
X. Ouyang et al. (May, 2020) [88]	Novel online attention module with a 3D CNN (convolutional neural network)	ROC curve, AUC, sensitivity, specificity, F1 score	Eight hospital COVID-19 patients’ CT data	No	87.50%	86.90%	90.10%	82.00%	0.944	NR
D.P. Fan et al. (Apr, 2020) [89]	Semi-supervised segmentation framework (propagation strategy)	Dice similarity coefficient, mean absolute error	COVID-19 CT segmentation dataset	Yes	NR	0.725	0.960	NR	NR	(Mean absolute error) MAE: Semi Inf.net = 0.082 Inf Net (Dice Similarity Coefficient) DSC = 0.064. DSC: Semi Inf.net = 0.682 Inf Net DSC = 0.739
X. Qian et al. (Dec, 2020) [90]	Deep learning made of two 2D CNN networks	TPR, TNR, TP, TN (true positive rate, true negative rate, true positive, true negative), false positive/negative error, false disease prediction error, ROC, AUC	Hospital information system data	Yes	95.21%	NR	NR	NR	NR	FP 2.83% and FN 4.15%
H.Y. Pei et al. (Mar, 21) [91]	A multi-point supervised training structure, implemented into MPS-Net (multi-point supervised network)	Dice similarity coefficient (DSC), sensitivity, specificity, and IOU	Not reported	Yes	NR	84.06%	84.06%	NR	NR	DSC = 0.8235 and IOU = 0.742
J. Wang et al. (May, 2020) [92]	Double 3D-ResNets using prior-attention strategy	AUC	Multiple hospitals’ CT images	Yes	NR	NR	NR	NR	97.3%	NR
D. Wu et al. (Oct, 2020) [93]	Hybrid weak label-based deep learning method. Based on UNet and EM algorithms.	Box plots, TPR, FPR, Mann–Whitney U test, Pearson correlation coefficient	International multi-retrospective studies’ data	No	NR	NR	NR	NR	NR	Severity segmentation, Pearson correlation coefficient of r = 0.825 (*p* < 0.001)
L. Li et al. (Mar, 2020) [94]	COVNet framework consists of RestNet50	AUC, sensitivity, and specificity, analysis of variance tests, and Chi-squared tests	Six hospitals’ retrospective and multicenter study data	No	NR	90%	96%	NR	0.96	NR
X. Wu et al. (Jul, 2020) [95]	Trained a multi-view fusion model based on the architecture of ResNet50.	AUC, Mann–Whitney U test, and Chi-square test	Three hospitals’ retrospective study data	Yes	Validation: 0.7, test: 0.760	Validation 0.730, test: 0.811	Validation0.615 test: 0.615		Validation: 0.732 test: 0.819	NR
K. Wang et al. (May, 2020) [96]	Correlation between the severity of chest infection, lymphocyte ratio, and SpO_2_ (blood oxygen saturation, usually in percentage)	Mean, standard deviation, median value interquartile range, case numbers, and percentages. Spearman’s test	114 confirmed COVID-19 patients’ retrospective study data	Yes	NR	NR	NR	NR	NR	CT results have a negative correlation with SpO_2_ (r = −0.446), and lymphocyte numbers (r = −0.780)
A. Oulefki et al. (Nov, 2020) [97]	Present an approach to enhance and segment images	Accuracy, sensitivity, F-measure, precision, MCC, Dice, Jacquard, and specificity	COVID-19 patient CT image dataset	Yes	0.98	0.71	0.99	0.73	NR	Precision = 0.723, MCC = 0.71, Dice Jacquard = 0.71 and specificity = 0.57
M. Pennisi et al. (May, 2021) [98]	Tiramisu architecture-based segmentation model and a fully convolutional DenseNet in a U-Net architecture	Chi-squared test, AUC, Sensitivity, specificity	Prospective study, 166 CT scans overall	Yes	0.84	0.93	93.5	NR	NR	NR
M. Polsinelli et al. (Dec, 2020) [99]	A light CNN based on SqueezeNet	Accuracy, sensitivity, specificity, precision, F1 score	Two different datasets	Yes	0.8503	NR	NR	NR	NR	Improvement of 3.2% in the first dataset arrangement and 2.1% in the second

**Table A4:** Summary of key research articles from last ten years using chest X-ray images (a radiographic method for the diagnosis of CAP), retrieved from IEEE Xplore, PubMed, and Science Direct. Latest research has been given preference. This table summarizes the methods for identifying pneumonia using CXRs. It also briefly presents the evaluation techniques used to test the given methods and the datasets used by each researcher. Finally, it can be extracted easily from the table if any given method has been compared with other related models or methods, and accuracy, sensitivity, specificity, AUC, and other result parameters have been given.

Author	Techniques	Evaluation Techniques	Data	Compared with Related Work	Results				
					Acc. (Accuracy)	Sen. (Sensitivity)	Spec. (Specificity)	AUC (Area under ROC Curve)	Other
V. Chouhan et al. (Jan, 2020) [100]	Transfer learning using AlexNet, DenseNet121, InceptionV3, resNet18, and GoogLeNet	Accuracy, AUC, ROC (receiver operating characteristic curve)	Dataset of Guangzhou Women and Children’s Medical Center	Yes	Ensemble model: 0.96395	NR (Not reported)	NR	Ensemble model: 99.34%	Recall 99.62%
Jilian D. Londono et al. (Dec, 2020) [101]	Deep CNN	Test positive predictive value, sensitivity, F1 score, accuracy, balanced accuracy geometric, and area under the ROC curve	ACT, China set, Montgomery, CRX8, CheXpert, and MIMIC datasets; COVID-19, BIMCV, ACT, and HM Hospital datasets.	No	0.915	0.874	NR	NR	NR
M. E. H. Chowdhary et al. (Jun, 2020) [102]	CNN (convolutional neural network) models	Comparison of the ROC curve for normal, COVID-19, and viral pneumonia	Kaggle database [30]	Yes	0.997, 0.979	0.997, 0.979	0.995, 0.988	NR	DenseNet201 outperforms other different deep CNN networks
X. Yu et al. (Jan, 2021) [103]	Deep learning using CGNet	Sensitivity, specificity, accuracy, precision, F1 score, ROC curves	Dataset 1: bacterial pneumonia. Dataset2: COVID-19-induced pneumonia	Yes	Dataset 1: 0.9872 dataset2: 0.99	Dataset 1: 1 Dataset2:0.98	Dataset 1: 0.9795 Dataset2: 1	NR	NR
P. Rajpurkar et al. (Dec, 2017) [104]	121-layer CNN called Chexnet	F1 Score	Chest X-ray 14	Yes	NR	NR	NR	NR	Chest X-ray 14 achieves state-of-the-art results
P. Chhikara et al. (Oct, 2019) [105]	Deep CNN model with transfer learning	Classification matrices, ROC curve	Database from Guangzhou Women and Children’s Medical Center	Yes	0.901	0.957	NR	NR	F1 score: 0.931 Precision 0.907
M. M. Ahsan et al. (Feb, 2021) [106]	Proposed and tested six modified deep learning models: 1. VGG16; 2. Inception ResNetV; 3. ResNet50; 4. MobileNetV2; 5. ResNet101; and 6. VGG19	Accuracy, precision, recall, F1 score, paired *t*-test	Study One: smaller, balanced dataset: obtained from the open-source repository. Study Two: larger, imbalanced dataset: obtained from the Kaggle COVID-19 chest X-ray dataset. Study Three: multiclass dataset	Yes	0.91	NR	NR	NR	Models like Inception ResNetV2 and VGG19 demonstrated an accuracy of 97% on both datasets
K.K. Singh et al. (Feb, 21) [107]	CNN using wavelet decomposition	Accuracy, sensitivity, and F1 measure	A total of 1439 images from the three classes are available	Yes	0.9583	0.9607	NR	NR	Precision = 0.956, F1 score = 0.9563
Ahishali et al. (Jun, 2200) [108]	Convolutional Support Estimator Network (CSEN)	Accuracy, sensitivity, specificity	Early-QaTa COVID-19	Yes	NR	0.97	0.997	NR	NR
C. J. Saul et al. (Apr, 2019) [109]	Deep learning architecture for the classification task	Accuracy	RSNA (Radiological Society of North America) dataset	Yes	0.7873	NR	NR	NR	NR
S. Yao et al. (Mar, 2020) [110]	GeminiNet to identify and localize, DetNet59 to capture deep features	Mean average precision, AUC, ROC	RSNA dataset	Yes	NR	NR	NR	NR	NR
E Rozenberg et al. (Apr, 2021) [111]	Neural network architecture uses Conditional random field layers	Intersection-over-union and intersection-over-region	RSNA dataset, NIH (National Institute of Health) chest X-ray dataset	Yes	Max IoU accuracy: 0.924+−0.06, max IoR accuracy = 0.935 + −0.07	NR	NR	NR	IoU accuracy = 0.918 ± 0.07, IOR accuracy = 0.933 ± 0.06
A. A. Saraiva et al. (Jan, 2019) [112]	Multilayer perceptron and CNN	Confusion matrix	CXR data set, Guangzhou Women and Children’s Medical Centre	Yes	94.40%	NR	NR	NR	NR
J.X. Wu et al. (Jun, 2020) [113]	Multilayer machine vision bases classifier	Mean recall, mean precision, mean accuracy, and mean F1 score	NIH chest X-ray database	No	0.8537	0.9868	NR	NR	Mean precision = 82.42%, mean F1 score = 0.8981
R. G. Babukarthik et al. (Sep, 2020) [114]	Genetic deep learning CNN	Accuracy, sensitivity, specificity, F1 score confusion matrix	Custom-built dataset from GitHub	Yes	0.9884	0.93–1	0.97	NR	NR
J. Zhang et al. (Mar, 21) [115]	Confidence-Aware Anomaly Detection (CAAD) model	AUC, sensitivity, specificity, accuracy	X-VIRAL and XCOVID datasets	Yes	NR	0.717	NR	83.61%	NR
G. Liang et al. (Apr, 20) [116]	A deep learning + residual thought + dilated convolution using the Keras framework	Precision, recall, F1 score, accuracy, AUC, ROC, confusion matrices	CXR dataset by Kermany et al. [117]	Yes	NR	0.967	NR	NR	F1 score = 92.7%.
O. Stephen et al. (Mar, 19) [118]	CNN model (Keras open-source deep learning framework) + tensor flow backend	Training accuracy	Retrospective pediatric patients between 1 and 5 years old	No	0.9531	NR	NR	NR	Training loss: 0.1288, validation loss: 0.1835
Y. Xu et al. (July, 21) [119]	MANet, segmentation model based on ResNet (Residual Network) classic CNNs with or without MA	Confusion matrix, accuracy, precision, recall, F1 score, attenuation heat maps	CXR dataset by Kermany et al. [117], Montgomery County and Shenzhen No. 3 People’s Hospital, and open public dataset on GitHub for COVID-19	Yes	0.9631	NR	NR	NR	ResNet50 accuracy = 96.32%
M. Yaseliani et al. (June, 2022)	Hybrid CNN, three classification approaches	Accuracy, precision, recall, specificity, and F1 score	CXR dataset by Kermany et al. [117]	No	98.55	NR	NR	NR	-
A. Chharia et al. (Feb, 2022)	De novo biologically inspired Conv-Fuzzy network is developed	Accuracy, precision, recall, F1 score	Kaggle CXR dataset, COVID-CXR dataset [120]	No	Binary class 97.47, multiclass 90.68	Binary 97.46, multiclass 90.67	Binary 97.46 multi-class 90.70	NR	-

**Table A5:** Summary of key research articles from the last ten years using lung ultrasound scan (LUS) (a radiographic method for the diagnosis of CAP), retrieved from IEEE Xplore, PubMed, and Science Direct. This table summarizes the methods for identifying pneumonia using lung ultrasound. It also briefly presents the evaluation techniques used to test the given methods and the datasets used by each researcher. Finally, it can be extracted easily from the table if any given method has been compared with other related models or methods, and accuracy, sensitivity, specificity, AUC, and other result parameters have been given.

Author	Techniques	Evaluation Methods	Data	Compared with Related Work	Results				
					Acc. (Accuracy)	Sen. (Sensitivity)	Spec. (Specificity)	AUC (Area under ROC Curve)	Other
MM.C. Ho et al. (Jul, 15) [121]	LUS scan features + chest X-ray + LUS	Mean, standard deviation values, numbers, and percentages	Three-year retrospective study data	Yes	0.9754	NR (Not reported)	NR	NR	NR
S. Roy et al. (May, 20) [122]	Deep network made from Spatial Transformer Network	The mean and standard deviation of the weighted F1 score, precision, and sensitivity	Italian COVID-19 Lung Ultrasound Database (ICLUS-DB)	Between various models and subsets of the original dataset	NR	NR	NR	NR	Reg-STN (Regression Spatial Transformer Network) performs the best amongst all baselines
Laura E. Ellington et al. (May, 17) [123]	A pediatrician’s clinical assessment and lung ultrasound	Sensitivity, specificity, AUC	A prospective study for primary respiratory complaints at the Instituto Nacional de Salud del Ni~no in Lima, Peru	Yes	NR	0.885	1	0.94	NR
S. Ottaviani et al. (Aug, 20) [124]	All patients had their lungs examined with HRCT and ultrasonography by separate operators who were blinded to each other’s results	Wilcoxon’s chi-square test, Kruskal–Wallis, correlation by the Spearman correlation coefficient.	Prospective single-center study	Yes	NR	NR	NR	NR	Correlation between the ultrasound score for B lines and the classification (*p* < 0.01) and percentage of lung involvement on chest HRCT: r = 0.935, *p* < 0.001
A. Reissig et al. (Oct, 12) [125]	LUS, reference test and follow-up performed	Sensitivity, specificity, likelihood ratio, Bland–Altman plots	Prospective, multicenter study: enrolled in 14 European centers.	Yes	NR	0.934	0.977	NR	For positive likelihood ratios = 40.5 (95% CI (confidence interval), 13.2-123.9), negative likelihood 0.07 (95% CI, 0.04–0.11)
J. Lovrenski et al. (2016) [126]	In one hour, LUS and auscultatory procedures were performed	McNemar’s test	Seven-month prospective study data	Yes	67/95	NR	NR	NR	Lung ultrasound showed a positive finding in more hemi-thoraces than auscultation (in children with clinically suspected pneumonia)
C. Biagi et al. (Dec, 18) [127]	LUS was performed in each child by a pediatrician without a patient chart and history	Cohen’s Kappa test, chi-square test, Mann–Whitney test, sensitivity, specificity, positive predictive value and negative predictive value, spearman’s Correlation coefficient	A prospective study performed at the Pediatric Emergency Unit of S. Orsola-Malpighi Hospital (Bologna, Italy) in association with the Pediatric Radiology Unit (2016–2017)	Yes	NR	1	0.839	92%	When only consolidation > 1 cm was considered consistent with pneumonia, the specificity of LUS increased to 98.4%, and the sensitivity decreased to 80.0%
L. Ambroggio et al. (Jun, 16) [128]	Four pediatric radiologists who were not aware of the patient’s condition were assessed on the CXR and LUS scans	Median and IQR for continuous variables and for categorical variables. For diagnosis, count and percentage, IRR, sensitivity, specificity, Kappa	A prospective cohort study of children with a CXR and LUS performed with or without a clinical diagnosis of pneumonia (1 May 2012, to 31 January 2014)	Yes	NR	NR	NR	NR	Pneumonia was clinically documented in 47 patients (36%)
G. Tan et al. (Jun, 20) [129]	Interstitial lung disease was used to evaluate the severity of COVID-19 on ultrasonography evaluated by the modified Buda scoring system.	Wilcoxon rank-sum tests, chi-square or Fisher exact tests for categorical variables, Pearson correlation	One-month prospective study data	Yes	NR	NR	NR	NR	Differences in ultrasonic features between COVID-19 and CAP were statistically significant
G. Muhammad et al. (Aug, 21) [130]	Model of a CNN (convolutional neural network)	Confusion matrix, ROC, AUC	POCUS (point-of-care ultrasound) dataset	Yes	0.866, 0.925	NR	NR	NR	NR
Vaishali P. Shah et al. (Feb, 13) [131]	Clinicians with 1 hour of focused training used ultrasonography	Likelihood ratios, sensitivity, and specificity with 95% CIs	A prospective observational cohort study in two urban ERs (emergency rooms)	Yes	NR	0.86	0.89	NR	Positive LR = 7.8 (95% CI, 5.0–12.4), negative LR = 0.2 (95% CI, 0.1–0.4)
Y. Wang et al. (Aug, 21)	SVM classifier on pleural line and B line features to determine three categories of COVID-19 pneumonia: moderate, severe, and critical	Correlation between features and disease severity, ROC, AUC, specificity, sensitivity	27 COVID-19 patients	No	NR	0.93	1	NR	ROC = 0.96

**Table A6:** Summary of key research articles from the last ten years using physiological parameters extracted using different sensors for the diagnosis of CAP, re-trieved from IEEE Xplore, PubMed, and Science Direct. This table summarizes the methods for identifying pneumonia and briefly presents the evaluation techniques used to test the given methods and the datasets used by each researcher. Finally, it can be extracted easily from the table if any given method has been compared with other related models, and the results are summarized.

Authors	Techniques	Input Parameters	Evaluation Methods	Data	Compared with Related Work	Results
W. Karlen et al. (Jul, 13) [132]	Pulse oximetry	PPG/pulse oximetry	ANOVA (analysis of variance) for the test dataset	Collected physiological data to calibrate the algorithms and evaluate the RR estimation performance	No	The mean RMS (root mean square) error (±1 SD (standard deviation)) of the smart fusion (3 ± 4.7 breaths/min)
Mei-Jing Ly et al. (Jan, 20) [133]	EEG (electroencephalogram), PPG (photoplethysmogram), stethoscope, smart dog	Sensors took vital signs	Pie charts	NR (not reported)	No	Most of the caregivers were satisfied
K. Mala et al. (Oct, 16) [134]	RR, HR (heart rate), SpO_2_, and body temperature	Sensors took vital signs	Experimental data into graphs	NR	No	Zero difference in vital sign measurement between the proposed and conventional devices
T. Salti et al. (Dec, 19) [135]	RR (respiratory rate), SpO2 (oxygen saturation in blood, usually given in percentage)	Sensors took vital signs	Shapiro–Wilk test, paired *t*-test, Pearson coefficients, Bland–Altman analysis	NR	Yes	Mean, standard deviation and 95 CI (confidence interval) for SpO_2_ = 97.2%, 1.3%, 96–98%; for RR = 18.6%, 5.4%, 14.4–22.7
Shih-Wen Chiu et al. (Dec, 14) [136]	CMOsGas sensor	Expired gas	Accuracy	Clinical experiment at Taipei Medical University, Taiwan.	Yes	100% accuracy to identify the microorganisms of Klebsiella, Pseudomonas aeruginosa, Staphylococcus aureus, and Candida from VAP-infected patients was achieved
S. Doulou et al. (Nov, 20) [137]	Novel optical biosensor (OB)	Sensor data	AUC (area under ROC curve), ROC (receiver operating characteristic curve), sensitivity, specificity,	A clinical study was conducted in four study sites in two phases	Yes, with the gold standard	Sensitivity = 83.3%, negative predictive value = 87.5%

## References

[1] Mathers C., Stevens G., Hogan D., Mahanani W.R., Ho J. (2017). Global and Regional Causes of Death: Patterns and Trends, 2000–2015. Disease Control Priorities: Improving Health and Reducing Poverty.

[2] McAllister D.A., Liu L., Shi T., Chu Y., Reed C., Burrows J., Adeloye D., Rudan I., Black R.E., Campbell H. (2019). Global, regional, and national estimates of pneumonia morbidity and mortality in children younger than 5 years between 2000 and 2015: A systematic analysis. Lancet Glob. Health.

[3] CME Info—Child Mortality Estimates. https://childmortality.org/.

[4] Grimwood K., Chang A.B. (2015). Long-term effects of pneumonia in young children. Pneumonia.

[5] Mackenzie G. (2016). The definition and classification of pneumonia. Pneumonia.

[6] Community-Acquired Pneumonia–Pulmonary Disorders–MSD Manual Professional Edition. https://www.msdmanuals.com/professional/pulmonary-disorders/pneumonia/community-acquired-pneumonia.

[7] Hospital Acquired Pneumonia–Pulmonology Advisor. https://www.pulmonologyadvisor.com/home/decision-support-in-medicine/pulmonary-medicine/hospital-acquired-pneumonia/.

[8] Averjanovaitė V., Saikalytė R., Cincilevičiūtė G., Kučinskaitė G., Mačiulytė D., Kontrimas A., Maksimaitytė V., Zablockis R., Danila E. (2018). Risk factors for early onset severe community-acquired pneumonia complications. Eur. Respir. J..

[9] Mbata G., Chukwuka C., Onyedum C., Onwubere B., Aguwa E. (2013). The role of complications of community acquired pneumonia on the outcome of the illness: A prospective observational study in a tertiary institution in Eastern Nigeria. Ann. Med. Health Sci. Res..

[10] Welte T., Torres A. (2012). Nathwani. Clinical and economic burden of community-acquired pneumonia among adults in Europe. Thorax.

[11] Levy M.L., Le Jeune I., Woodhead M.A., Macfarlane J.T., Lim W.S. (2010). Primary care summary of the British Thoracic Society guidelines for the management of community acquired pneumonia in adults: 2009 Update. Prim. Care Respir. J..

[12] Overview of Community-Acquired Pneumonia in Adults–UpToDate. https://www.uptodate.com/contents/overview-of-community-acquired-pneumonia-in-adults.

[13] Reynolds A.R. (1903). Pneumonia: The new ‘captain of the men of death.’: Its increasing prevalence and the necessity of methods for its restriction. J. Am. Med. Assoc..

[14] Witzenrath M., Kuebler W.M. (2020). Pneumonia in the face of COVID-19. Am. J. Physiol. Lung Cell. Mol. Physiol..

[15] Tricco A.C., Lillie E., Zarin W., O’Brien K.K., Colquhoun H., Levac D., Moher D., Peters M.D.J., Horsley T., Weeks L. (2018). PRISMA extension for scoping reviews (PRISMA-ScR): Checklist and explanation. Ann. Intern. Med..

[16] Colquhoun H.L., Levac D., O’Brien K.K., Straus S., Tricco A.C., Perrier L., Kastner M., Moher D. (2014). Scoping reviews: Time for clarity in definition, methods, and reporting. J. Clin. Epidemiol..

[17] Munn Z., Peters M.D.J., Stern C., Tufanaru C., McArthur A., Aromataris E. (2018). Systematic review or scoping review? Guidance for authors when choosing between a systematic or scoping review approach. BMC Med. Res. Methodol..

[18] Torres A., Serra-Batlles J., Ferrer A., Jiménez P., Celis R., Cobo E., Rodriguez-Roisin R. (1991). Severe community-acquired pneumonia. Epidemiology and prognostic factors. Am. Rev. Respir. Dis..

[19] Melbye H., Hvidsten D., Holm A., Nordbø A., Brox J. (2004). The course of C-reactive protein response in untreated upper respiratory tract infection. Br. J. Gen. Pract..

[20] Mandell L.A., Wunderink R.G., Anzueto A., Bartlett J.G., Campbell G.D., Dean N.C., Dowell S.F., File T.M., Musher D.M., Niederman M.S. (2007). Infectious Diseases Society of America/American Thoracic Society Consensus Guidelines on the management of community-acquired pneumonia in adults. Clin. Infect. Dis..

[21] Andronikou S., Goussard P., Sorantin E. (2017). Computed tomography in children with community-acquired pneumonia. Pediatr. Radiol..

[22] Orso D., Ban A., Guglielmo N. (2018). Lung ultrasound in diagnosing pneumonia in childhood: A systematic review and meta-analysis. J. Ultrasound.

[23] Bourcier J.E., Braga S., Garnier D. (2016). Lung Ultrasound Will Soon Replace Chest Radiography in the Diagnosis of Acute Community-Acquired Pneumonia. Curr. Infect. Dis. Rep..

[24] Yadav K.K., Awasthi S., Parihar A. (2017). Lung Ultrasound is Comparable with Chest Roentgenogram for Diagnosis of Community-Acquired Pneumonia in Hospitalised Children. Indian J. Pediatr..

[25] Sergunova K., Bazhin A., Masri A., Vasileva Y.N., Semenov D., Kudryavtsev N., Panina O.Y., Khoruzhaya A., Zinchenko V., Akhmad E. (2021). Chest MRI of patients with COVID-19. Magn. Reson. Imaging.

[26] Jackson K., Butler R., Aujayeb A. (2021). Lung ultrasound in the COVID-19 pandemic. Postgrad. Med. J..

[27] Franquet T. (2001). Imaging of pneumonia: Trends and algorithms. Eur. Respir. J..

[28] Nambu A. (2014). Imaging of community-acquired pneumonia: Roles of imaging examinations, imaging diagnosis of specific pathogens and discrimination from noninfectious diseases. World J. Radiol..

[29] Zhang D., Ren F., Li Y., Na L., Ma Y. (2021). Pneumonia Detection from Chest X-ray Images Based on Convolutional Neural Network. Electronics.

[30] COVID-19 Radiography Database|Kaggle. https://www.kaggle.com/tawsifurrahman/covid19-radiography-database.

[31] Chest X-ray Images (Pneumonia)|Kaggle. https://www.kaggle.com/paultimothymooney/chest-xray-pneumonia.

[32] Durant A., Nagdev A. (2010). Ultrasound detection of lung hepatization. West J. Emerg. Med..

[33] McDermott C., Łącki M., Sainsbury B., Henry J., Filippov M., Rossa C. (2021). Sonographic Diagnosis of COVID-19: A Review of Image Processing for Lung Ultrasound. Front. Big Data.

[34] Wanasinghe T., Bandara S., Madusanka S., Meedeniya D., Bandara M., Diez I.D.L.T. (2024). Lung Sound Classification with Multi-Feature Integration Utilizing Lightweight CNN Model. IEEE Access.

[35] Kanwal K., Khalid S.G., Asif M., Zafar F., Qurashi A.G. (2024). Diagnosis of Community-Acquired pneumonia in children using photoplethysmography and Machine learning-based classifier. Biomed. Signal Process. Control.

[36] (2020). QuickStats: Death Rates* from Influenza and Pneumonia† Among Persons Aged ≥65 Years, by Sex and Age Group—National Vital Statistics System, United States, 2018. MMWR Morb. Mortal. Wkly. Rep..

[37] Ostapchuk M., Roberts D.M., Haddy R. (2004). Community-Acquired Pneumonia in Infants and Children. Am. Fam. Physician.

[38] Li W., Ding C., Yin S. (2015). Severe pneumonia in the elderly: A multivariate analysis of risk factors. Int. J. Clin. Exp. Med..

[39] Coronavirus Pandemic (COVID-19)–the Data–Statistics and Research–Our World in Data. https://ourworldindata.org/coronavirus-data.

[40] Dallas J., Kollef M. (2009). Severe hospital-acquired pneumonia: A review for clinicians. Curr. Infect. Dis. Rep..

[41] Al-Omari B., McMeekin P., Allen A.J., Akram A.R., Graziadio S., Suklan J., Jones W.S., Lendrem B.C., Winter A., Cullinan M. (2021). Systematic review of studies investigating ventilator associated pneumonia diagnostics in intensive care. BMC Pulm. Med..

[42] Wunderink R.G., Waterer G.W. (2014). Community-Acquired Pneumonia. N. Engl. J. Med..

[43] Christ-Crain M., Opal S.M. (2010). Clinical review: The role of biomarkers in the diagnosis and management of community-acquired pneumonia. Crit. Care.

[44] Povoa P. (2008). Serum markers in community-acquired pneumonia and ventilator-associated pneumonia. Curr. Opin. Infect. Dis..

[45] Ye X., Xiao H., Chen B., Zhang S. (2015). Accuracy of Lung Ultrasonography versus Chest Radiography for the Diagnosis of Adult Community-Acquired Pneumonia: Review of the Literature and Meta-Analysis. PLoS ONE.

[46] Stokes K., Castaldo R., Federici C., Pagliara S., Maccaro A., Cappuccio F., Fico G., Salvatore M., Franzese M., Pecchia L. (2022). The use of artificial intelligence systems in diagnosis of pneumonia via signs and symptoms: A systematic review. Biomed. Signal Process. Control.

[47] Gentilotti E., De Nardo P., Cremonini E., Górska A., Mazzaferri F., Canziani L.M., Hellou M.M., Olchowski Y., Poran I., Leeflang M. (2022). Diagnostic accuracy of point-of-care tests in acute community-acquired lower respiratory tract infections. A systematic review and meta-analysis. Clin. Microbiol. Infect..

[48] Heidari A., Navimipour N.J., Unal M., Toumaj S. (2022). The COVID-19 epidemic analysis and diagnosis using deep learning: A systematic literature review and future directions. Comput. Biol. Med..

[49] Sajjad Z. (2003). Neuro-Imaging Facilities in Pakistan. J. Pak. Med. Assoc..

[50] Li G., Li X., Song X., Zeng Y. (2022). Edge Blockchain Construction Efficiency Maximization for COVID-19 Detection in a Body Area Network. IEEE Access.

[51] Gilbert S. (2024). The EU passes the AI Act and its implications for digital medicine are unclear. npj Digit. Med..

[52] Keane P.A., Topol E.J. (2018). With an eye to AI and autonomous diagnosis. npj Digit. Med..

[53] Zhou K., Gattinger G. (2024). The Evolving Regulatory Paradigm of AI in MedTech: A Review of Perspectives and Where We Are Today. Ther. Innov. Regul. Sci..

[54] Li Z., Li X., Zhu Z., Zeng S., Wang Y., Wang Y., Li A. (2019). Signal Analysis of Electrocardiogram and Statistical Evaluation of Myocardial Enzyme in the Diagnosis and Treatment of Patients with Pneumonia. IEEE Access.

[55] Gadsby N.J., Russell C.D., McHugh M.P., Mark H., Morris A.C., Laurenson I.F., Hill A.T., Templeton K.E. (2016). Comprehensive molecular testing for respiratory pathogens in community-acquired pneumonia. Clin. Infect. Dis..

[56] Borgohain R., Baruah S. (2017). Development and testing of zno nanorods based biosensor on model gram-positive and gram-negative bacteria. IEEE Sens. J..

[57] Koster M.J., Broekhuizen B.D., Minnaard M.C., Balemans W.A., Hopstaken R.M., de Jong P.A., Verheij T.J. (2013). Diagnostic properties of C-reactive protein for detecting pneumonia in children. Respir. Med..

[58] Sordé R., Falcó V., Lowak M., Domingo E., Ferrer A., Burgos J., Puig M., Cabral E., Len O., Pahissa A. (2011). Current and potential usefulness of pneumococcal urinary antigen detection in hospitalized patients with community-acquired pneumonia to guide antimicrobial therapy. Arch. Intern. Med..

[59] Yang S.-L., Wen Y.-H., Wu Y.-S., Wang M.-C., Chang P.-Y., Yang S., Lu J.-J. (2020). Diagnosis of Pneumocystis pneumonia by real-time PCR in patients with various underlying diseases. J. Microbiol. Immunol. Infect..

[60] Holter J.C., Müller F., Bjørang O., Samdal H.H., Marthinsen J.B., Jenum P.A., Ueland T., Frøland S.S., Aukrust P., Husebye E. (2015). Etiology of community-acquired pneumonia and diagnostic yields of microbiological methods: A 3-year prospective study in Norway. BMC Infect. Dis..

[61] Ito A., Yamamoto Y., Ishii Y., Okazaki A., Ishiura Y., Kawagishi Y., Takiguchi Y., Kishi K., Taguchi Y., Shinzato T. (2021). Evaluation of a novel urinary antigen test kit for diagnosing Legionella pneumonia. Int. J. Infect. Dis..

[62] Lu Y., Ling G., Qiang C., Ming Q., Wu C., Wang K., Ying Z. (2011). PCR diagnosis of Pneumocystis pneumonia: A bivariate meta-analysis. J. Clin. Microbiol..

[63] Elemraid M.A., Sails A.D., Thomas M.F., Rushton S.P., Perry J.D., Eltringham G.J., Spencer D.A., Eastham K.M., Hampton F., Gennery A.R. (2013). Pneumococcal diagnosis and serotypes in childhood community-acquired pneumonia. Diagn. Microbiol. Infect. Dis..

[64] Esteves F., Calé S., Badura R., de Boer M., Maltez F., Calderón E., van der Reijden T., Márquez-Martín E., Antunes F., Matos O. (2015). Diagnosis of Pneumocystis pneumonia: Evaluation of four serologic biomarkers. Clin. Microbiol. Infect..

[65] Jiang Y., Wang H., Hao S., Chen Y., He J., Liu Y., Chen L., Yu Y., Hua S. (2020). Digital PCR is a sensitive new technique for SARS-CoV-2 detection in clinical applications. Clin. Chim. Acta.

[66] Xiao D., Zhao F., Lv M., Zhang H., Zhang Y., Huang H., Su P., Zhang Z., Zhang J. (2012). Rapid identification of microorganisms isolated from throat swab specimens of community-acquired pneumonia patients by two MALDI-TOF MS systems. Diagn. Microbiol. Infect. Dis..

[67] Wang Y., Yan W., Wang Y., Xu J., Ye C. (2018). Rapid, sensitive and reliable detection of Klebsiella pneumoniae by label-free multiple cross displacement amplification coupled with nanoparticles-based biosensor. J. Microbiol. Methods.

[68] Medjo B., Atanaskovic-Markovic M., Radic S., Nikolic D., Lukac M., Djukic S. (2014). Mycoplasma pneumoniae as a causative agent of community-acquired pneumonia in children: Clinical features and laboratory diagnosis. Ital. J. Pediatr..

[69] Edin A., Eilers H., Allard A. (2020). Evaluation of the Biofire Filmarray Pneumonia panel plus for lower respiratory tract infections. Infect. Dis..

[70] Banerjee A., Ray S., Vorselaars B., Kitson J., Mamalakis M., Weeks S., Baker M., Mackenzie L.S. (2020). Use of Machine Learning and Artificial Intelligence to predict SARS-CoV-2 infection from Full Blood Counts in a population. Int. Immunopharmacol..

[71] Tsai C., Tang K., Cheng M., Liu T., Huang Y., Chen C., Yu H. (2020). Use of saliva sample to detect C-reactive protein in children with pneumonia. Pediatr. Pulmonol..

[72] Omran A., Ali M., Saleh M.H., Zekry O. (2018). Salivary C-reactive protein and mean platelet volume in diagnosis of late-onset neonatal pneumonia. Clin. Respir. J..

[73] Patrucco F., Gavelli F., Ravanini P., Daverio M., Statti G., Castello L.M., Andreoni S., Balbo P.E. (2019). Use of an innovative and non-invasive device for virologic sampling of cough aerosols in patients with community and hospital acquired pneumonia: A pilot study. J. Breath Res..

[74] Minnaard M.C., van de Pol A.C., de Groot J.A.H., De Wit N.J., Hopstaken R.M., van Delft S., Goossens H., Ieven M., Lammens C., Little P. (2015). The added diagnostic value of five different C-reactive protein point-of-care test devices in detecting pneumonia in primary care: A nested case-control study. Scand. J. Clin. Lab. Investig..

[75] Kosasih K., Abeyratne U.R., Swarnkar V., Triasih R. (2015). Wavelet Augmented Cough Analysis for Rapid Childhood Pneumonia Diagnosis. IEEE Trans. Biomed. Eng..

[76] Chen H., Yuan X., Pei Z., Li M., Li J. (2019). Triple-Classification of Respiratory Sounds Using Optimized S-Transform and Deep Residual Networks. IEEE Access.

[77] Porter P., Brisbane J., Abeyratne U., Bear N., Wood J., Peltonen V., Della P., Smith C., Claxton S. (2020). Diagnosing community-acquired pneumonia via a smartphone-based algorithm: A prospective cohort study in primary and acute-care consultations. Br. J. Gen. Pract..

[78] McCollum E.D., Park D.E., Watson N.L., Fancourt N.S.S., Focht C., Baggett H.C., Brooks W.A., Howie S.R.C., Kotloff K.L., Levine O.S. (2020). Digital auscultation in PERCH: Associations with chest radiography and pneumonia mortality in children. Pediatr. Pulmonol..

[79] Rao A., Ruiz J., Bao C., Roy S. (2018). Tabla: A Proof-of-Concept Auscultatory Percussion Device for Low-Cost Pneumonia Detection. Sensors.

[80] Imran A., Posokhova I., Qureshi H.N., Masood U., Riaz M.S., Ali K., John C.N., Hussain I., Nabeel M. (2020). AI4COVID-19: AI enabled preliminary diagnosis for COVID-19 from cough samples via an app. Inform. Med. Unlocked.

[81] Tripathy R.K., Dash S., Rath A., Panda G., Pachori R.B. (2022). Automated Detection of Pulmonary Diseases from Lung Sound Signals Using Fixed-Boundary-Based Empirical Wavelet Transform. IEEE Sens. Lett..

[82] Fraiwan M., Fraiwan L., Khassawneh B., Ibnian A. (2021). A dataset of lung sounds recorded from the chest wall using an electronic stethoscope. Data Brief.

[83] Lai Y., Li G., Wu D., Lian W., Li C., Tian J., Ma X., Chen H., Xu W., Wei J. (2020). 2019 novel coronavirus-infected pneumonia on CT: A feasibility study of few-shot learning for computerized diagnosis of emergency diseases. IEEE Access.

[84] Wang G., Liu X., Li C., Xu Z., Ruan J., Zhu H., Meng T., Li K., Huang N., Zhang S. (2020). A Noise-Robust Framework for Automatic Segmentation of COVID-19 Pneumonia Lesions from CT Images. IEEE Trans. Med. Imaging.

[85] Wang Z., Liu Q., Dou Q. (2020). Contrastive Cross-Site Learning with Redesigned Net for COVID-19 CT Classification. IEEE J. Biomed. Health Inf..

[86] Wang Q., Yang D., Li Z., Zhang X., Liu C. (2020). Deep regression via multi-channel multi-modal learning for pneumonia screening. IEEE Access.

[87] Kang H., Xia L., Yan F., Wan Z., Shi F., Yuan H., Jiang H., Wu D., Sui H., Zhang C. (2020). Diagnosis of Coronavirus Disease 2019 (COVID-19) with Structured Latent Multi-View Representation Learning. IEEE Trans. Med Imaging.

[88] Ouyang X., Huo J., Xia L., Shan F., Liu J., Mo Z., Yan F., Ding Z., Yang Q., Song B. (2020). Dual-Sampling Attention Network for Diagnosis of COVID-19 from Community Acquired Pneumonia. IEEE Trans. Med. Imaging.

[89] Fan D.-P., Zhou T., Ji G.-P., Zhou Y., Chen G., Fu H., Shen J., Shao L. (2020). Inf-Net: Automatic COVID-19 Lung Infection Segmentation from CT Images. IEEE Trans. Med. Imaging.

[90] Qian X., Fu H., Shi W., Chen T., Fu Y., Shan F., Xue X. (2020). M3Lung-Sys: A Deep Learning System for Multi-Class Lung Pneumonia Screening from CT Imaging. IEEE J. Biomed. Health Inf..

[91] Pei H.Y., Yang D., Liu G.R., Lu T. (2021). MPS-net: Multi-point supervised network for ct image segmentation of COVID-19. IEEE Access.

[92] Wang J., Bao Y., Wen Y., Lu H., Luo H., Xiang Y., Li X., Liu C., Qian D. (2020). Prior-Attention Residual Learning for More Discriminative COVID-19 Screening in CT Images. IEEE Trans. Med. Imaging.

[93] Wu D., Gong K., Arru C.D., Homayounieh F., Bizzo B., Buch V., Ren H., Kim K., Neumark N., Xu P. (2020). Severity and Consolidation Quantification of COVID-19 from CT Images Using Deep Learning Based on Hybrid Weak Labels. IEEE J. Biomed. Health Inf..

[94] Li L., Qin L., Xu Z., Yin Y., Wang X., Kong B., Bai J., Lu Y., Fang Z., Song Q. (2020). Using Artificial Intelligence to Detect COVID-19 and Community-acquired Pneumonia Based on Pulmonary CT: Evaluation of the Diagnostic Accuracy. Radiology.

[95] Wu X., Hui H., Niu M., Li L., Wang L., He B., Yang X., Li L., Li H., Tian J. (2020). Deep learning-based multi-view fusion model for screening 2019 novel coronavirus pneumonia: A multicentre study. Eur. J. Radiol..

[96] Wang K., Kang S., Tian R., Zhang X., Wang Y. (2020). Imaging manifestations and diagnostic value of chest CT of coronavirus disease 2019 (COVID-19) in the Xiaogan area. Clin. Radiol..

[97] Oulefki A., Agaian S., Trongtirakul T., Laouar A.K. (2021). Automatic COVID-19 lung infected region segmentation and measurement using CT-scans images. Pattern Recognit..

[98] Pennisi M., Kavasidis I., Spampinato C., Schinina V., Palazzo S., Salanitri F.P., Bellitto G., Rundo F., Aldinucci M., Cristofaro M. (2021). An Explainable AI System for Automated COVID-19 Assessment and Lesion Categorization from CT-scans. Artif. Intell. Med..

[99] Polsinelli M., Cinque L., Placidi G. (2020). A light CNN for detecting COVID-19 from CT scans of the chest. Pattern Recognit. Lett..

[100] Chouhan V., Singh S.K., Khamparia A., Gupta D., Tiwari P., Moreira C., Damaševičius R., de Albuquerque V.H.C. (2020). A novel transfer learning based approach for pneumonia detection in chest X-ray images. Appl. Sci..

[101] Arias-Londono J.D., Gomez-Garcia J.A., Moro-Velazquez L., Godino-Llorente J.I. (2020). Artificial Intelligence applied to chest X-ray images for the automatic detection of COVID-19. A thoughtful evaluation approach. IEEE Access.

[102] Chowdhury M.E.H., Rahman T., Khandakar A., Mazhar R., Kadir M.A., Bin Mahbub Z., Islam K.R., Khan M.S., Iqbal A., Al Emadi N. (2020). Can AI help in screening Viral and COVID-19 pneumonia?. IEEE Access.

[103] Yu X., Wang S.H., Zhang Y.D. (2021). CGNet: A graph-knowledge embedded convolutional neural network for detection of pneumonia. Inf. Process. Manag..

[104] Rajpurkar P., Irvin J., Zhu K., Yang B., Mehta H., Duan T., Ding D., Bagul A., Langlotz C., Shpanskaya K. CheXNet: Radiologist-Level Pneumonia Detection on Chest X-rays with Deep Learning. November 2017. http://arxiv.org/abs/1711.05225.

[105] Chhikara P., Singh P., Gupta P., Bhatia T. (2020). Deep convolutional neural network with transfer learning for detecting pneumonia on chest X-rays. Advances in Intelligent Systems and Computing.

[106] Ahsan M., Ahad T., Soma F.A., Paul S., Chowdhury A., Luna S.A., Yazdan M.M.S., Rahman A., Siddique Z., Huebner P. (2021). Detecting SARS-CoV-2 from chest X-ray using artificial intelligence. IEEE Access.

[107] Singh K.K., Singh A. (2021). Diagnosis of COVID-19 from chest X-ray images using wavelets-based depthwise convolution network. Big Data Min. Anal..

[108] Ahishali M., Degerli A., Yamac M., Kiranyaz S., Chowdhury M.E.H., Hameed K., Hamid T., Mazhar R., Gabbouj M. (2020). Advance Warning Methodologies for COVID-19 using Chest X-ray Images. IEEE Access.

[109] Saul C.J., Urey D.Y., Taktakoglu C.D. (2019). Early Diagnosis of Pneumonia with Deep Learning. arXiv.

[110] Yao S., Chen Y., Tian X., Jiang R. (2020). GeminiNet: Combine Fully Convolution Network with Structure of Receptive Fields for Object Detection. IEEE Access.

[111] Rozenberg E., Freedman D., Bronstein A.A. (2021). Learning to Localize Objects Using Limited Annotation, with Applications to Thoracic Diseases. IEEE Access.

[112] Saraiva A., Santos D., Costa N., Sousa J., Ferreira N., Valente A., Soares S. Models of Learning to Classify X-ray Images for the Detection of Pneumonia using Neural Networks. Proceedings of the 12th International Joint Conference on Biomedical Engineering Systems and Technologies (BIOSTEC 2019).

[113] Wu J.X., Chen P.Y., Li C.M., Kuo Y.C., Pai N.S., Lin C.H. (2020). Multilayer Fractional-Order Machine Vision Classifier for Rapid Typical Lung Diseases Screening on Digital Chest X-ray Images. IEEE Access.

[114] Babukarthik R.G., Adiga V.A.K., Sambasivam G., Chandramohan D., Amudhavel A.J. (2020). Prediction of COVID-19 using genetic deep learning convolutional neural network (GDCNN). IEEE Access.

[115] Zhang J., Xie Y., Pang G., Liao Z., Verjans J., Li W., Sun Z., He J., Li Y., Shen C. (2021). Viral Pneumonia Screening on Chest X-rays Using Confidence-Aware Anomaly Detection. IEEE Trans. Med. Imaging.

[116] Liang G., Zheng L. (2020). A transfer learning method with deep residual network for pediatric pneumonia diagnosis. Comput. Methods Programs Biomed..

[117] Kermany D.S., Goldbaum M., Cai W., Valentim C.C.S., Liang H., Baxter S.L., McKeown A., Yang G., Wu X., Yan F. (2018). Identifying Medical Diagnoses and Treatable Diseases by Image-Based Deep Learning. Cell.

[118] Stephen O., Sain M., Maduh U.J., Jeong D.U. (2019). An Efficient Deep Learning Approach to Pneumonia Classification in Healthcare. J. Healthc. Eng..

[119] Xu Y., Lam H.K., Jia G. (2021). MANet: A two-stage deep learning method for classification of COVID-19 from Chest X-ray images. Neurocomputing.

[120] Cohen J.P., Morrison P., Dao L., Roth K., Duong T.Q., Ghassemi M. (2020). COVID-19 Image Data Collection: Prospective Predictions Are the Future. J. Mach. Learn. Biomed. Imaging.

[121] Ho M.C., Ker C.R., Hsu J.H., Wu J.R., Dai Z.K., Chen I.C. (2015). Usefulness of lung ultrasound in the diagnosis of community-acquired pneumonia in children. Pediatr. Neonatol..

[122] Roy S., Menapace W., Oei S., Luijten B., Fini E., Saltori C., Huijben I., Chennakeshava N., Mento F., Sentelli A. (2020). Deep Learning for Classification and Localization of COVID-19 Markers in Point-of-Care Lung Ultrasound. IEEE Trans. Med. Imaging.

[123] Ellington L.E., Gilman R.H., Chavez M.A., Pervaiz F., Marin-Concha J., Compen-Chang P., Riedel S., Rodriguez S.J., Gaydos C., Hardick J. (2017). Lung ultrasound as a diagnostic tool for radiographically-confirmed pneumonia in low resource settings. Respir. Med..

[124] Ottaviani S., Franc M., Ebstein E., Demaria L., Lheure C., Debray M., Khalil A., Crestani B., Borie R., Dieudé P. (2020). Lung ultrasonography in patients with COVID-19: Comparison with CT. Clin. Radiol..

[125] Reissig A., Copetti R., Mathis G., Mempel C., Schuler A., Zechner P., Aliberti S., Neumann R., Kroegel C., Hoyer H. (2012). Lung ultrasound in the diagnosis and follow-up of community-acquired pneumonia: A prospective, multicenter, diagnostic accuracy study. Chest.

[126] Lovrenski J., Petrović S., Balj-Barbir S., Jokić R., Vilotijević-Dautović G. (2016). Stethoscope vs. ultrasound probe–which is more reliable in children with suspected pneumonia?. Acta Med. Acad..

[127] Biagi C., Pierantoni L., Baldazzi M., Greco L., Dormi A., Dondi A., Faldella G., Lanari M. (2018). Lung ultrasound for the diagnosis of pneumonia in children with acute bronchiolitis. BMC Pulm. Med..

[128] Ambroggio L., Sucharew H., Rattan M.S., O’Hara S.M., Babcock D.S., Clohessy C., Steinhoff M.C., Macaluso M., Shah S.S., Coley B.D. (2016). Lung Ultrasonography: A Viable Alternative to Chest Radiography in Children with Suspected Pneumonia?. J. Pediatr..

[129] Tan G., Lian X., Zhu Z., Wang Z., Huang F., Zhang Y., Zhao Y., He S., Wang X., Shen H. (2020). Use of Lung Ultrasound to Differentiate Coronavirus Disease 2019 (COVID-19) Pneumonia from Community-Acquired Pneumonia. Ultrasound Med. Biol..

[130] Muhammad G., Hossain M.S. (2021). COVID-19 and Non-COVID-19 Classification using Multi-layers Fusion From Lung Ultrasound Images. Inf. Fusion.

[131] Shah V.P., Tunik M.G., Tsung J.W. (2013). Prospective evaluation of point-of-care ultrasonography for the diagnosis of pneumonia in children and young adults. JAMA Pediatr..

[132] Karlen W., Raman S., Ansermino J.M., Dumont G.A. (2013). Multiparameter respiratory rate estimation from the photoplethysmogram. IEEE Trans. Biomed. Eng..

[133] Lyu M.J., Yuan S.M. (2020). Cloud-Based Smart Dog Music Therapy and Pneumonia Detection System for Reducing the Difficulty of Caring for Patients with Dementia. IEEE Access.

[134] Mala K., Kumar B.M., Vignesh R., Kumar K.M. A wearable diagnostic device to combat children’s pneumonia. Proceedings of the GHTC 2016–IEEE Global Humanitarian Technology Conference: Technology for the Benefit of Humanity, Conference Proceedings.

[135] El Salti T., Sykes E.R., Zajac W., Abdullah S., Khoja S. (2019). NewPneu: A Novel Cost Effective mHealth System for Diagnosing Childhood Pneumonia in Low-Resource Settings. Proceedings of the 2019 IEEE 10th Annual Information Technology, Electronics and Mobile Communication Conference, IEMCON 2019.

[136] Chiu S.-W., Wang J.-H., Chang K.-H., Chang T.-H., Wang C.-M., Chang C.-L., Tang C.-T., Chen C.-F., Shih C.-H., Kuo H.-W. (2014). A fully integrated nose-on-a-chip for rapid diagnosis of ventilator-associated pneumonia. IEEE Trans. Biomed. Circuits Syst..

[137] Doulou S., Leventogiannis K., Tsilika M., Rodencal M., Katrini K., Antonakos N., Kyprianou M., Karofylakis E., Karageorgos A., Koufargyris P. (2020). A novel optical biosensor for the early diagnosis of sepsis and severe COVID-19: The PROUD study. BMC Infect. Dis..

